# Responses of Mast Cells to Pathogens: Beneficial and Detrimental Roles

**DOI:** 10.3389/fimmu.2021.685865

**Published:** 2021-06-15

**Authors:** Mariela Jiménez, Daniel Cervantes-García, Laura E. Córdova-Dávalos, Marian Jesabel Pérez-Rodríguez, Claudia Gonzalez-Espinosa, Eva Salinas

**Affiliations:** ^1^ Laboratory of Immunology, Department of Microbiology, Universidad Autónoma de Aguascalientes, Aguascalientes, Mexico; ^2^ Cátedras CONACYT, National Council of Science and Technology, Mexico City, Mexico; ^3^ Department of Pharmacobiology, Centro de Investigación y de Estudios Avanzados (Cinvestav), Unidad Sede Sur, Mexico City, Mexico

**Keywords:** mast cells, phagocytosis, extracellular traps, mast cell mediators, pathology development participation, signaling pathways

## Abstract

Mast cells (MCs) are strategically located in tissues close to the external environment, being one of the first immune cells to interact with invading pathogens. They are long living effector cells equipped with different receptors that allow microbial recognition. Once activated, MCs release numerous biologically active mediators in the site of pathogen contact, which induce vascular endothelium modification, inflammation development and extracellular matrix remodeling. Efficient and direct antimicrobial mechanisms of MCs involve phagocytosis with oxidative and non-oxidative microbial destruction, extracellular trap formation, and the release of antimicrobial substances. MCs also contribute to host defense through the attraction and activation of phagocytic and inflammatory cells, shaping the innate and adaptive immune responses. However, as part of their response to pathogens and under an impaired, sustained, or systemic activation, MCs may contribute to tissue damage. This review will focus on the current knowledge about direct and indirect contribution of MCs to pathogen clearance. Antimicrobial mechanisms of MCs are addressed with special attention to signaling pathways involved and molecular weapons implicated. The role of MCs in a dysregulated host response that can increase morbidity and mortality is also reviewed and discussed, highlighting the complexity of MCs biology in the context of host-pathogen interactions.

## Introduction

Described by Paul Ehrlich in 1878 and widely studied in the context of allergy, the mast cells (MCs) are cellular components of the immune system that perform crucial functions in innate and adaptive immune responses (1). MCs contain cytoplasmic granules that store a plethora of preformed mediators, such as heparin, histamine and enzymes, mainly chymase, tryptase and carboxypeptidase A, which are released upon cell activation. Depending on the stimulus, MCs can also *de novo* synthesize eicosanoids, such as leukotrienes (LTs), prostaglandins (PGs) and platelet activation factor, as well as a wide variety of cytokines, chemokines, and growth factors (2). Several of these compounds prompt vasodilation, an increase in vascular permeability and recruitment of inflammatory cells during the allergic process and the antimicrobial response.

**Figure 1:**
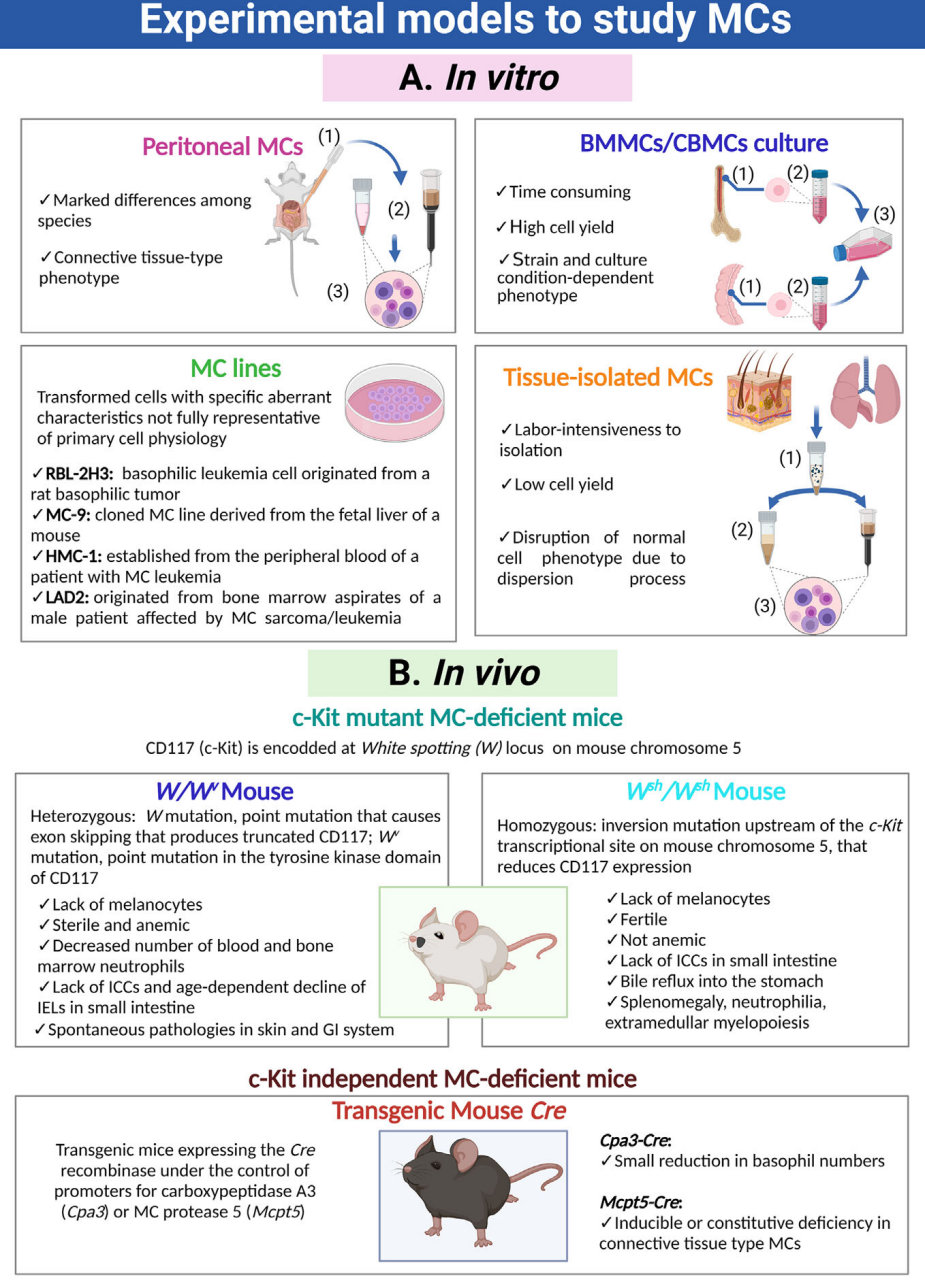
Cellular and animal models utilized to investigate MCs. **(A)** Distinct MC preparations and cultures used for *in vitro* approaches. Purification of peritoneal MCs requires (1) peritoneal lavages, (2) purification through density gradients or magnetic beads coupled to specific antibodies and (3) final recovery of cells. Generation of bone marrow-derived MCs or cord blood-derived MCs requires (1) the isolation and disruption of the primary organ, (2) purification of immature precursors and (3) culture of those precursors for a prolonged period of time in the presence of specific cytokines and growth factors. Isolation of tissue-resident MCs is a process that requires (1) fragmentation of the organ and gentle enzymatic digestion, (2) purification of MCs utilizing density gradients, cell sorting or magnetic beads coupled to specific antibodies and (3) recovery of MCs. **(B)** Main animal models to analyze the role of MCs *in vivo*, indicating their phenotypic abnormalities. MC, mast cell; ICCs, interstitial cells of Cajal; IELs, intraepithelial lymphocytes TCRγδ; GI, gastro-intestinal.

Different experimental models are used to study MC biology and its participation in physiological and pathological processes (**Figure 1**). *In vitro* studies of MCs are predominantly performed using MCs isolated from the peritoneal cavity of mice and rats (3–5), or rodent or human MCs obtained by cultures from bone marrow progenitors (BMMC), umbilical cord blood progenitors (CBMC) or embryonic stem cells (6–9). Immortalized MC lines from rodent (RBL-2H3, MC-9) and human (HMC-1, LAD2) origin have been also developed and are commonly used (5, 10, 11). In addition, MCs can be isolated from peripheral tissues through enzymatic digestion and enrichment processes (12). MC transcriptome changes depending on the tissue from which cells are obtained or whether they are or not subjected to culture conditions (13, 14). In this sense, the identification of tissue-specific expressed genes arises the possibility to study individual cell population within the tissue, circumventing the necessity of extensive MC purification (13, 14). *In vivo* studies of MCs were detonated with the discovery of c-Kit mutant MC-deficient mice (most used are *W/W^v^*, *W^sh^/W^sh^*) and the development of c-Kit independent MC-deficient mice strains (*Cpa3-Cre* and *Mcpt5-Cre*) (15–19). These animal models permit to evaluate the role of MCs in particular conditions, since they can be reconstituted by adoptive transfer of cultured MCs obtained from congenic wild-type or transgenic or knock-out mice (20). Each experimental approach has its own limitations to consider when interpreting or extrapolating the results (**Figure 1**).

## Origin, Location, Heterogeneity, and Physiological Functions

Early observations led to consider MCs as components of connective tissue derived from undifferentiated mesenchymal cells. The hematopoietic origin of MCs in mice and humans was demonstrated in 1977 and 1994, respectively, when it was shown that these cells were derived from bone marrow (BM) progenitor cells (21, 22). Recently, the use of hematopoietic fate mapping tools in mice revealed that MCs initially derive from yolk sac precursors in the embryo but are progressively replaced by definitive MCs at later stages of development (23). During embryogenesis, early erythro-myeloid progenitors (EMP)-derived MCs firstly populate most tissues, but are later replaced in most connective tissues by late EMP-derived MCs with exception of adipose tissue and pleural cavity; finally, fetal hematopoietic stem cells (HSC)-derived MCs populate the mucosa (24). After birth, these embryonic MCs continue their development into mature MCs. While evidence support that mucosal MCs depend on adult HSCs for their replacement, connective MCs do not. Specifically, MC progenitors in skin expand locally to form clonal colonies and mature MCs are self-maintained independent of BM, except during the inflammatory process in which there is an influx of new BM-progenitors that proliferate to form new colonies (25). In humans, a single MC-committed progenitor derived directly from the pluripotent stem cell CD34^+^, c-Kit^+^ was described (26). This progenitor was sensitive to stem cell factor (SCF), the ligand of c-Kit receptor, and can be detected in BM, peripheral blood, and peripheral tissues (27). In mice, three MC-committed progenitors were described, two of them in BM which were derived directly either from a multipotent progenitor or from a common myeloid progenitor, and the other one in the spleen (28). The MC-committed progenitors circulate in the vascular system as immature progenitor cells and complete their maturation when homing within tissues and are exposed to the influence of characteristic factors of each tissue. In humans, in response to several cytokines such as interleukin (IL)-3, IL-4, IL-9 and IL-10, they stop expressing CD34 and the IL-3Rα chain (CD123) and begin expressing higher levels of the high-affinity receptor for IgE (FcϵRI) and c-Kit (29–32). Besides ILs, SCF derived from tissue-resident stromal cells also regulate MC differentiation, maturation, and survival (33). The importance of the tissue microenvironment in MC maturation is evidenced when MCs are transferred from one anatomical site to another, as they change their phenotype (20, 34).

MCs reside near to blood vessels and nerve endings in almost all vascularized tissues, being especially abundant in the skin and the mucosal tissues, which are sites exposed to the external environment and the gateway of pathogens (35). Mature MCs constitute a very heterogeneous cell population both in humans and rodents, showing differences in number, distribution, type of expressed proteases, proteoglycans and vasoactive amines, surface receptors and growth factors that drive their differentiation, as summarized in **Tables 1** and **2** (2, 36–59). This plasticity enables MCs to respond to local specific signals, in normal and pathological conditions.

**Table 1:** Main characteristics of MC types described in rodents.

	MMCs	CTMCs	References
**Distribution**	Nasal and pulmonary mucosa, intestinal lamina propria	Skin and peritoneum	(2, 36, 37)
**Size**	7–12 μm (rat)	17-22 μm (rat)	(2, 38, 39)
**Granules**	Few granules and with variable size (rat)	Many granules and with little size variability (rat)	(40)
**Behavior**	Migratory (rat)	Nonmigratory (rat)	(41)
**Proteases**	MCPT-1, MCPT-2 (chymases) (mouse) MCPT-2, MCPT-5 (chymases) (rat)	MCPT-3, MCPT-4 (chymases) MCPT-5 (elastase) MCPT-6 (tryptase) MCPT-7 (tryptase) CPA3 (mouse) MCPT-1 (chymase) CPA3 (rat)	(37, 41, 42)
**Amines**	Histamine (low amount) Serotonin	Histamine (high amount) Serotonin	(37, 42, 43)
**Proteoglycans**	Chondroitin sulfate E (mouse) Chondroitin sulfate di-B, A, E (rat)	Heparin (mouse) Heparin Chondroitin sulfate E (rat)	(37, 41)
**T-cell dependence in *in vivo* development**	Yes	No	(38, 42, 44)
**Cytokine needed to *in vitro* proliferation**	IL-3	IL-4 in the presence of IL-3 (mouse) SCF in presence of IL-3 (rat)	(43, 45–47)
**Sensitive to C48/80**	No	Yes	(40, 48–50)
**Activated by SP**	No	Yes	(49, 51)
**Inhibited by sodium cromoglycate**	No	Yes	(42, 48, 50, 52)

MC, mast cell; MMCs, Mucosal-type mast cells; CTMCs, Connective tissue–type mast cells; MCPT, mast cell protease; CPA, carboxypeptidase; C48/80, compound 48/80; SP, substance P; SCF, stem cell factor; IL, interleukin.

**Table 2:** Main characteristics of MC types described in humans.

	MC_T_	MC_TC_	MC_C_	References
**Distribution**	Nasal and small intestinal mucosa, alveoli	Skin, small intestinal submucosa	Submucosa, mucosa of the stomach, submucosa of the small intestine, mucosa of the colon	(41, 53, 54)
**Size**	9.2 µm	9.9 µm	Not reported	(55)
**Proteases**	Tryptase	Tryptase, Chymase, CPA3, Catepsine G, Granzyme B	Chymase	(54, 56)
**Amines**	Histamine	Histamine	Not reported	(37)
**Proteoglycans**	Chondroitin sulfate A, E and heparin	Chondroitin sulfate A, E and heparin	Not reported	(37, 41, 57)
**T-cell dependence**	Yes	No	Not reported	(58)
**Sensitive to C48/80**	No	Yes	Not reported	(42)
**Activated by SP**	No	Yes	Not reported	(42)
**Inhibited by sodium cromoglycate**	Yes	No	Not reported	(42, 59)

MC, mast cells; MC_T_, mast cell tryptase-type; MC_TC_, mast cell tryptase and chymase-type; MC_C_, mast cell chymase-type; CPA, carboxypeptidase; C48/80, compound 48/80; SP, substance P.

MCs play key roles in the modulation of diverse physiological processes (60–64). MCs participate in wound healing and bone remodeling, since in their absence both processes are impaired (65–68). MCs store preformed molecules that improve fibroblast and epithelial cell proliferation, leukocyte recruitment and collagen synthesis in damaged tissue, such as tryptase (69–74) and chymase (75, 76). Besides wound healing, angiogenesis and lymphangiogenesis are also influenced by MCs (77–80). They produce several angiogenic mediators, such as histamine, tryptase, matrix metalloproteinase (MMP)-2 and -9, chymase, vascular endothelial growth factor A, platelet-derived growth factor and fibroblast growth factor (77, 81–86). Moreover, MCs are closely residents of nerve endings (87, 88), executing a bidirectional crosstalk with nerve fibers (89–92). MCs also regulate cardiovascular and renal systems (93–96), and participate in cancer control (97, 98).

In addition, a wealth of evidence supports the protective role of MCs during infectious processes, although, under certain circumstances MC response to microbial encounter may lead to harmful conditions in the host. This dual effect of MC activation in the response to pathogens will be revised in detail in the next sections, firstly reviewing the antimicrobial mechanisms that generate protection in the host, i.e. MC beneficial roles, and finally, those conditions in which the response of the cell to the microbial stimulus induces damage in the host, considered as MC detrimental roles.

## Antimicrobial Roles of Mast Cells

Due to their strategic location and the expression of a wide panel of receptors, MCs represent a sentinel system for the detection of invading pathogens with the capacity to generate an immediate response against them (35, 63, 99). Traditionally, MCs have been categorized as starters of the innate response against pathogens, however they can also promote the activation of adaptive response by: i) cytokine secretion, such as tumor necrosis factor (TNF)-α, that induces the migration of dendritic cells (DC) to draining lymph nodes or T cell proliferation; ii) exosome secretion containing class II major histocompatibility complex (MHC) and co-stimulatory molecules; iii) the formation of immunological synapses with DC that facilitate the transfer of endosomal content and other molecules between both cells; and iv) presenting antigens and directly activating antigen-experienced T cell (100). This latter action is highly interesting, because it places MCs as important direct participants in the initiation of adaptive immunity. For example, co-culture of T cells with BMMCs caused T cell proliferation by FcϵRI-dependent and FcϵRI-independent mechanisms, being the latter dependent on the MC secretion of TNF-α (101). In another study, human psoriatic skin biopsies showed an important infiltrate of IL-22^+^ CD4^+^ T cells that were found in contact with MCs (102). *In vitro*, human MCs were observed forming immunologic synapses with CD4^+^ T lymphocytes, inducing the expansion of Th22 and IL-22/interferon (IFN)-γ-producing Th cells (102). Finally, after FcϵRI or Toll-like receptor (TLR)4 activation, murine and human MCs upregulated the synthesis of molecules associated with antigen presentation, enabling the autologous memory T cell activation (103, 104).

Participation of MCs in responses against microbes occurs by direct interactions with microorganisms, and by recognition of products from the damaged tissue. MC responses can produce a direct antimicrobial effect and the recruitment and activation of effector cells (35, 63, 99). Direct interactions between MCs and pathogens, as in other immune cells, mainly occur *via* the activation of pattern-recognition-receptors (PRRs), while antibody-mediated interactions occur through Fc receptors. Evidence collected from distinct MC preparations has shown that they express all the main families of PRRs (105). Members of the TLR family (TLR1, 2, 3, 4, 5, 6, 7, 8 and 9) have been detected by real-time PCR in murine MCs (106, 107). Also, nucleotide-binding oligomerization domain (NOD)-like and retinoic acid-inducible gene-I-like receptor families (108), together with the C-type lectin receptors and the Mas-related G protein-coupled receptors have been identified in cultured and/or in freshly isolated MCs from mice and humans (109).

The different roles played by MCs in the elimination of pathogens can be classified as follows: phagocytosis, formation of MC-derived extracellular traps (MCETs), and secretion of preformed and newly synthesized mediators. In the following sections, information about each one of those processes, together with the known signal transduction pathways involved, is presented.

### Phagocytosis

**Figure 2:**
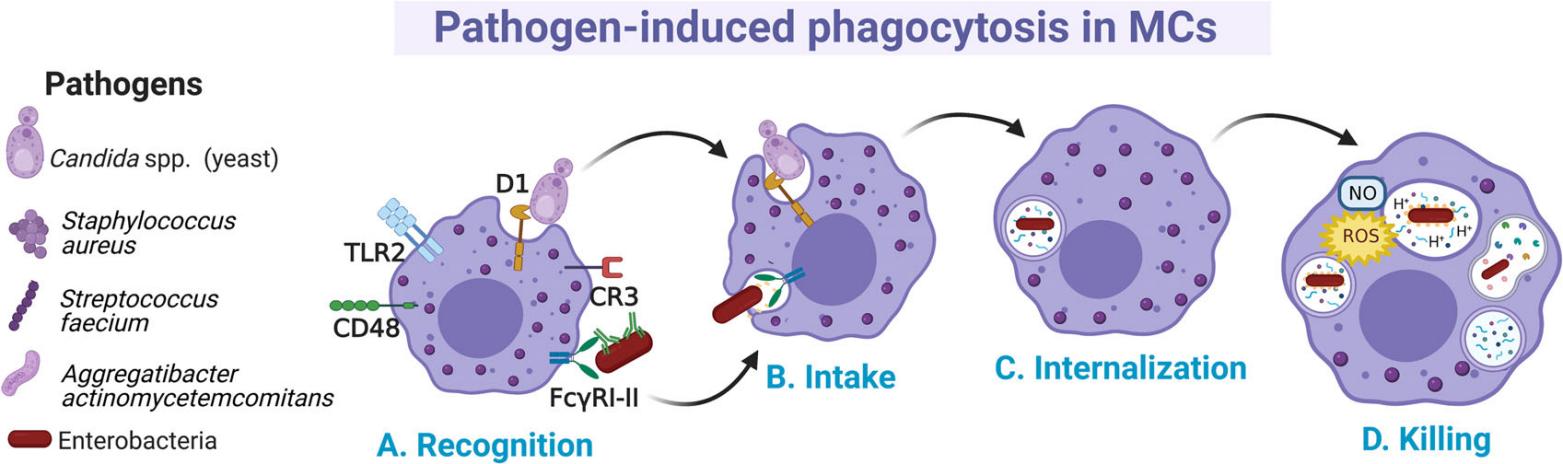
Main events occurring during pathogen phagocytosis in MCs. **(A)** Several membrane receptors bind free or opsonized pathogens. After the recognition phase, **(B)** signal transduction pathways involving cytoskeletal re-arrangements lead to the intake of pathogens and **(C)** internalization into a phagocytic vesicle named phagosome. **(D)** Pathogens are killed by the fusion of phagosome with lysosomes and by the generation of nitric oxide (NO) and reactive oxygen species (ROS) as part of the respiratory burst.

Distinct MC preparations have shown the capacity to internalize microbes by canonical cellular processes, although the details of all involved receptors and signaling cascades have not been fully described (110) (**Figure 2A**). In MCs, several phagocytosis-inducing receptors have been described. Some of them activate the process through the direct recognition of pathogen-associated molecular patterns, such as TLR2 or the mannosylated protein CD48, whereas other receptors (like CR3 and FcγRI and FcγRII enable phagocytosis of opsonin-bound pathogens (111–113). Early evidence about phagocytosis in MCs was described in an opsonin-dependent manner in rat peritoneal MCs, where it was shown that sheep erythrocytes covered with IgG and C3b were actively phagocytosed (114). Later works showed that the phagocytosis in MCs also represented a mechanism of pathogen elimination. Human CBMCs engulfed and destroyed Gram-negative (*Citrobacter freundii* clinical isolate (CI)125, *Klebsiella pneumoniae* CI128) and Gram-positive (*Streptococcus faecium* CI126, *Staphylococcus aureus* CI127) bacteria (115). This broad recognizing capacity was proposed to be mediated by specific antibodies and complement proteins. Once bacteria were attached to the surface of the cell, protoplasmic protrusions started to surround them (**Figure 2B**), and then, internalized bacteria could be observed in vacuoles together with a time-dependent decrease in their viability (**Figure 2C**).

MCs can internalize pathogens expressing the mannose-binding FimH from type I fimbriae such as *Escherichia coli*, with subsequent bactericidal activity through the production of reactive oxygen species (ROS), mainly the superoxide anion (**Figure 2D**) (116, 117). The capacity of MCs to phagocytose microbes could be specific for certain MC populations or conditions, since it was also reported that some preparations of MCs, such as mice BMMCs were unable to phagocytose *Salmonella typhimurium* and *Listeria monocytogenes* (118). Recognition of FimH-expressing bacteria by MCs was mediated by the glycosylphosphatidylinositol-anchored molecule CD48 (115). When phagocytosis occurs, it is assumed that phagocytic vacuoles are acidified, as the treatment with ammonium chloride reduced the microbicidal activity (116, 117) (**Figure 2D**). On the other hand, human CBMCs were shown to internalize *S. aureus* through a process mediated by CD48 and TLR2 receptors and dependent on alive bacteria and a functional cytoskeleton (119). In this case, *S. aureus* internalization was associated with increased survival of bacteria and the extracellular release of IL-8 and TNF-α. Nevertheless, in serum-free conditions the mechanism of FimH-expressing *E. coli* uptake by BMMCs was mediated through cellular caveolae, since intracellular bacteria were contained in chambers surrounded by caveolin (120). CD48 was co-localized with caveolin in the plasma membrane of the cell. This endocytic route of *E. coli* internalization was distinct from the classical endosome-lysosome pathway, which might allow bacteria to remain in a viable state (121). Similarly, it was reported that internalization of *Aggregatibacter actinomycetemcomitans* by murine BMMCs happens at different rates depending on whether opsonization was present or absent, being higher without opsonization (122). Whether A. *actinomycetemcomitans* is killed once internalized under each condition needs to be further investigated.

MCs also phagocyte and kill yeasts, which indicate that they may have an important role against fungal infections (123). Members of the family *Candida* spp. are common inhabitants of human skin and mucosal cavities, and they behave as opportunistic pathogens in superficial and systemic infections (124). Rat peritoneal MCs had discrete phagocytic activity on heat-killed *Candida albicans*; while yeast opsonization with rat serum increased the percentage of phagocytizing cells. Nevertheless, the percentage of killing of non-opsonized yeast was notably higher than those opsonized, which might suggest that extracellular killing capacity is more important than the one achieved intracellularly (113). The phagocytosis rate of *C. albicans* diminished when TLR2-deficient BMMCs were employed or an antagonistic antibody against Dectin-1 was used. Moreover, the killing capacity of murine BMMCs against *C. albicans* was found dependent on intracellular nitric oxide (NO) production (125).

A few studies have shown that once MCs have phagocytosed microbes, they can process microbial antigens for presentation to T cells. Using an assay in which a well-characterized T cell epitope was expressed within bacteria as a fusion protein, it was demonstrated that MCs are capable of processing bacterial antigens for presentation through class I MHC molecules to T cell hybridomas (126). Recently, MCs have been shown to take up and process both soluble and particulate antigens in an IgG opsonization- and IFN-γ-independent manner, however, while OVA or particulate antigens can be internalized through different pathways, viral antigen capture by MCs was mainly mediated through clathrin and caveolin-dependent endocytosis but not through phagocytosis or micropinocytosis (104). MC secretory granules were used for antigen processing, although the specific proteases involved were not described and require further research. When MCs were stimulated with IFN-γ, they expressed HLA-DR, HLA-DM as well as co-stimulatory molecules, which enable them to activate an antigen-specific recall response of CD4^+^ Th1 cells (104).

### Extracellular Traps

**Figure 3:**
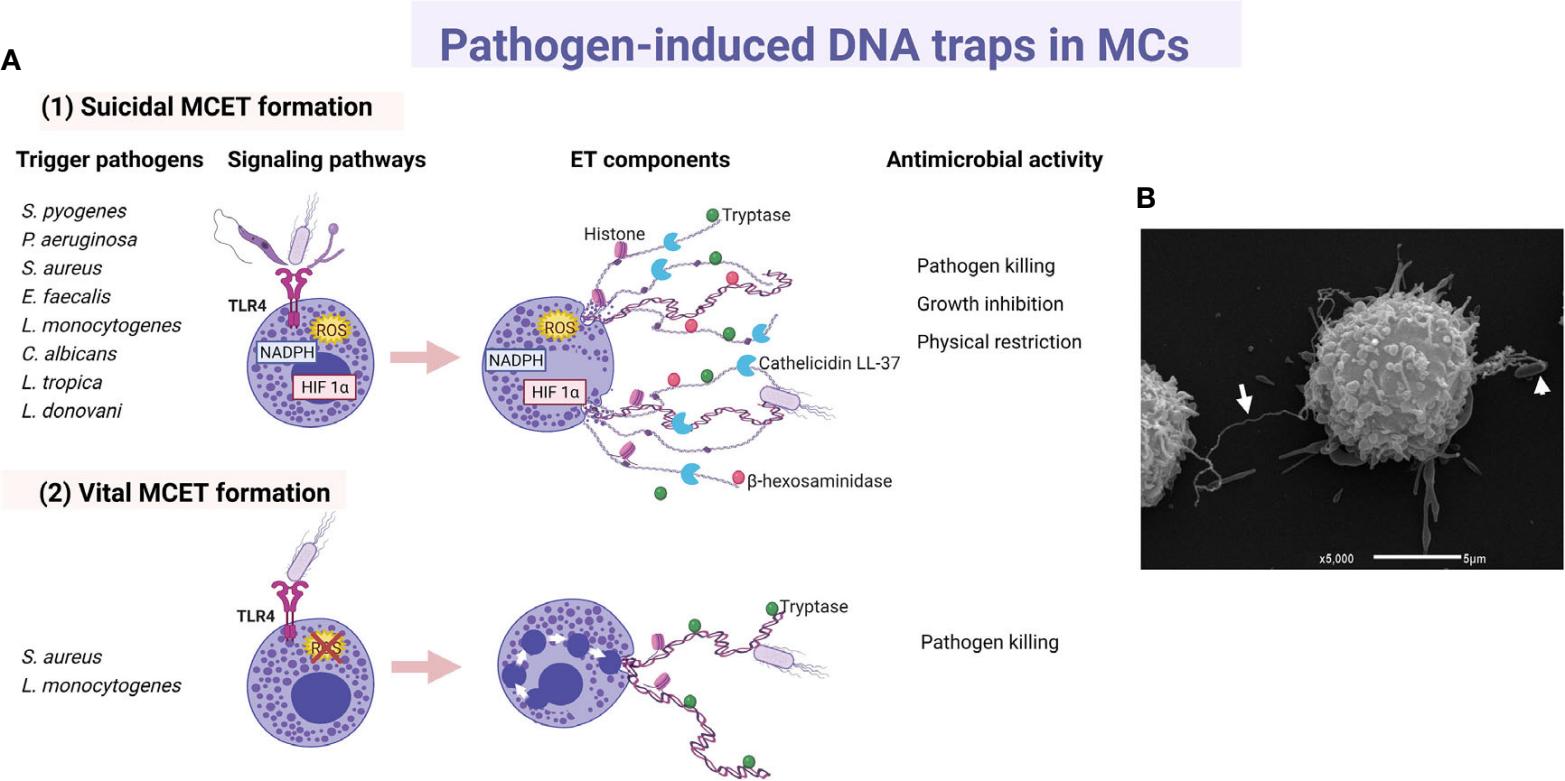
Main characteristics of pathogen-induced MC extracellular traps. **(A)** Principal triggers, activated signaling cascades and components of (1) suicidal and (2) vital MC extracellular traps (MCET) leading to distinct antimicrobial activity. **(B)** Scan electron micrograph of MCET (white arrow) emerging from a bone marrow-derived mast cell in the presence of *E. coli* (white arrowhead). ET, extracellular traps; HIF, hypoxia-inducible factor.

Since 2003, a few studies proposed direct and phagocytosis-independent antimicrobial activity of MCs against bacteria, although the precise mechanism was unclear. The cathelicidin LL-37, a broad-spectrum antimicrobial peptide (AMP) stored in MC granules, was implicated in the antimicrobial mechanism of the cell against group A *Streptococcus* (GAS), proposing that its activity could be due to intracellular (after phagocytosis) or extracellular mechanisms (127). Furthermore, supernatants from cultured MCs were able to kill *Citrobacter rodentium*, indicating a possible extracellular antibacterial effect consistent with the cell capacity to produce AMPs (128). In 2008, four years after the description of extracellular trap (ET) formation by neutrophils (NETs) (129), it was demonstrated that MCs produced extracellular structures like NETs (named as MCETs) with antimicrobial activity (130). Those studies showed that the extracellular death of *Streptococcus pyogenes* (M23 serotype GAS) by MCs depended on the formation of MCETs, which consisted of a chromatin-DNA backbone decorated with histones, and specific granule proteins, such as tryptase and LL-37, that ensnared and killed bacteria. MCET formation was dependent on the nicotinamide adenine dinucleotide phosphate (NADPH) oxidase activity and occurred 15 minutes after exposure of MCs to the bacteria. The inhibition of *S. pyogenes* growth was unaffected by treatment with the phagocytosis inhibitor cytochalasin D, ruling out the possibility that antimicrobial activity was mediated through the phagocytic uptake of *S. pyogenes* by the cells; although a closeness between both elements, the bacteria and the MC, was required. For the first time, MCET formation was described in HMC-1 cells and murine BMMCs as an antimicrobial mechanism in which DNA backbone embedded with granule components and histones forms a physical trap that catches pathogens into a microenvironment highly rich in antimicrobial molecules (**Figure 3**).

ET formation by MCs was later described in response to other GAS strain (131), or to other extracellular bacteria. For example, by HMC-1 in contact with *Pseudomonas aeruginosa* (130), HMC-1 or BMMCs co-cultured with *S. aureus* (132), or BMMCs infected with *Enterococcus faecalis* (133). Bacteria entrapped in MCETs were killed (132, 133). Although the cathelicidin LL-37 has been designated as an important weapon in the antimicrobial activity of MCs against *E. faecalis* (133), its direct activity as part of MCET structure still needs to be investigated. In good correlation, M1 protein of GAS was an important contributor to the MCET response in HMC-1 cell infection, but at the same time it conferred resistance to MCET-dependent killing of the bacteria, at least in part by binding/sequestration of the cathelicidin LL-37 (134). Concerning intracellular bacteria, the cell line HMC-1 stimulated with *L. monocytogenes* also released MCETs that contain histone, tryptase and β-hexosaminidase (135). ET formation in response to *L. monocytogenes* was also a NADPH- and ROS-dependent process and, interestingly, the inhibition of the bacterial growth was partly due to β-hexosaminidase. The role of β-hexosaminidase in MCETs still requires to be elucidated.

As aforementioned, most studies in mouse MCs or human MC cell lines about MCET formation describe a ROS‐dependent process, that resembles neutrophil cell death involving ETs (suicidal ET formation), a phenomenon that occurs through chromatin decondensation and disruption of the nuclear membrane (see **Figure 3A**1) (136). Interestingly, cathelicidin LL-37 can reach the nucleus and disrupt the nuclear membrane during NET generation in human and murine neutrophils (137). In this context, cultured human LAD2 cells treated with a high concentration of exogenous LL-37 released nucleic acids extracellularly, suggesting that LL-37 is permeabilizing both nuclear and plasma membranes; nevertheless, no ET-like structures were released (138). As LL-37 can disrupt membranes both in bacterial and normal eukaryotic cells (139, 140), the role of LL-37 in the formation of MCETs through the alteration of cellular membranes remains to be elucidated. Recently, using a flow cytometry assay, it was described that *L. monocytogenes*, and to a lesser extent *S. aureus*, induced DNA externalization without intracellular ROS production in human primary MCs (141). Induction of DNA release by *L. monocytogenes* occurred in live human MCs, and the process was associated with a low level of cell death and the presence of tryptase in extracellular DNA (see **Figure 3A**2). A similar type of vital ET release had been described in neutrophils in response to *S. aureus*, in which the release of DNA occurred by fusion of DNA‐containing vesicles with the plasma membrane (142). Although more research is needed, the rapid and vital release of MCETs more adequately matches the long-living nature of these tissue-resident mature cells.

MCs express different PRRs and produce inflammatory mediators traditionally involved in the antiviral, antifungal and antiparasitic response in other cells (62, 105, 143). Nevertheless, few studies have investigated the participation of MCETs in host protective response against these non-bacterial pathogens. Concerning fungi, human CBMCs and HMC-1 cells released MCETs decorated with tryptase upon *C. albicans* stimulation (144). Although ET formation increased over the time of fungal infection, it affected only a very low percentage of cells. *C. albicans* was ensnared in DNA backbone, but in contrast to results reported in bacteria, fungal viability was not affected by MCETs as shown by DNase treatment assays. In accordance, MCETs might be contributing to the physical restriction of the fungal pathogen. On the other hand, promastigotes of *Leishmania tropica* (causing cutaneous Leishmaniasis) and *Leishmania donovani* (causing visceral Leishmaniasis) triggered ET release from mouse peritoneal MCs and RBL-2H3 cell line, the greatest effect being in response to the last parasite (145). These MCETs were composed of DNA, histones and tryptase, and apart from killing the promastigotes they might physically restrict the parasite dissemination (145). As tryptase has been involved in the killing of other parasites, such as *Toxoplasma* tachyzoites (146), it would be interesting to investigate its role in *Leishmania* promastigotes death induced by MCETs.

Many questions are still unanswered regarding the formation of MCETs and its role on MC responses to pathogens; among them, whether MCETs might restrict the inflammatory response by breaking down cytokines and chemokines, as described in NETs (147). In this context, *in vitro* assays showed that MC tryptase and chymase could cleave a lower number of cytokines and chemokines than neutrophil proteases (148–150). Interestingly, when combining both MC proteases, three of the most potent Th2 cytokines (thymic stromal lymphopoietin, IL-18 and IL-33) were cleaved (149), indicating that *in vivo* they might exert a potent negative feedback loop or a regulatory role on anti-parasitic immunity.

### Activation of MCs: Release of Pre-Formed and Newly Synthesized Mediators

MCs release immunoregulatory compounds in a specific and intensity-dependent fashion (82, 151). The best-characterized ones are the pre-formed mediators stored in secretory lysosomes (granules), such as histamine, proteases, TNF-α, serotonin and heparin, among others. Secretion of those mediators can occur in a massive event known as anaphylactic degranulation, which is highly dependent on intracellular Ca^2+^ increase and cytoskeletal re-arrangements (152). Degranulation involves the fusion of granule membrane to plasmatic membrane and the extrusion of almost all granule content in few minutes (152). On the other hand, pre-synthesized mediators can also be secreted by a process named piecemeal degranulation, that implies the gradual emptiness of granule content without apparent fusion of granule membrane with the plasma membrane, by a yet poorly described mechanism [Reviewed in (152)]. Also, the triggering of different receptors leads to *de novo* synthesis and secretion of lipid mediators by enzymes localized in plasma membrane, and the activation of transcription factors that induce the synthesis of mRNAs encoding cytokines, chemokines, angiogenic and growth factors. *De novo* synthesized cytokines and chemokines seem to be secreted by budding vesicles from the Golgi apparatus utilizing elements of the constitutive secretory pathway (63, 152), and, recently, secretion of exosomes containing regulatory molecules has also been described in MCs (reviewed in 153). Anaphylactic degranulation occurs through compound exocytosis within 15 to 90 seconds upon cell activation when triggered with a high intensity stimulus (such as the crosslinking of FcϵRI receptor), while piecemeal degranulation can take up to 30 minutes after stimulation of TLR4 receptor (99, 154, 155). On the other hand, the production of *de novo*-synthesized mediators can take from few minutes (arachidonic acid derivatives) to several hours (cytokines or growth factors). The best described mechanism of activation of MC is that triggered by the high intensity activation of the FcϵRI receptor. Antigen-dependent crosslinking of the IgE molecules bound to FcϵRI receptors causes the activation and autophosphorylation of Lyn and Fyn kinases. In turn, those kinases phosphorylate the immunotyrosine-activation-motifs located in the γ and β subunits of the receptor, creating docking sites for the amplifying kinase Syk. This event initiates a complex signaling cascade that leads to degranulation, synthesis of derivatives of arachidonic acid and activation of transcription factors that will give origin to cytokine mRNAs (156, reviewed in 157). Interestingly, a new mechanism of MC degranulation was described in 2015, and was named antibody-dependent degranulatory synapse (ADDS). This process was mediated by crosslinking of FcϵRI or FcγRIIA receptors by cell-bound IgE or IgG and it resulted in a polarized and sustained release or exposure of the granule content at the contact surface between both cells. The signalling pathways activated in ADDS involved tyrosine and the phosphorylation of the adapter protein LAT (linker for activation of T cells), together with the clearance of cortical actin (146).

In this section, we will review the preformed and *de novo*-synthetized mediators released by MCs in response to bacteria, viruses, parasites, and fungi, making emphasis on their antimicrobial activity.

#### Bacteria

In 1996, it was demonstrated the crucial role of MCs against acute bacterial infections. Echtenacher et al. showed that MC-deficient mice were significantly more sensitive to experimental acute bacterial peritonitis induced by cecal ligation and puncture (CLP) than normal mice of the same strain (158). Intraperitoneal reconstitution of MC-deficient mice with matured and differentiated BMMCs before peritonitis induction protected animals from its harmful effects. Nevertheless, the administration of anti-TNF-α antibodies immediately after CLP suppressed these protective effects. Simultaneously, it was reported an increased number of alive *K. pneumoniae* in MC-deficient mice after their intraperitoneal or intranasal inoculation, compared to that found in wild-type animals (159). These results revealed the role of MCs in the elimination of the bacteria, which is dependent on their activation by FimH, the production of TNF-α and the subsequent neutrophil chemoattraction. MCs were mainly related to an early (15 min) peak of TNF-α production after antigen administration (160). Using MC protease (MCPT)4-deficient mice with CLP of moderate severity, it was demonstrated that MCPT-4 enhanced survival of animals, at least in part by degrading peritoneal TNF-α at the initial stage of the infection that subsequently avoided an excessive recruitment of neutrophils to peritoneal cavity (161). The protective role of MCs in acute bacterial infection was further demonstrated using another model of genetically modified MC mouse, such as C57BL/6 *tg/tg*, that shows a diminution in the number of MCs in the skin and the peritoneal cavity due to a mutation that affects the expression of the microphthalmia-associated transcription factor (162). MCs also played important roles in the elimination of bacteria in other tissues, such as during the early stage of otitis media caused by *Haemophilus influenzae* (163), as well as in pneumonia caused by *Mycoplasma pulmonis* (164), decreasing the seriousness of the pathology.

**Figure 4:**
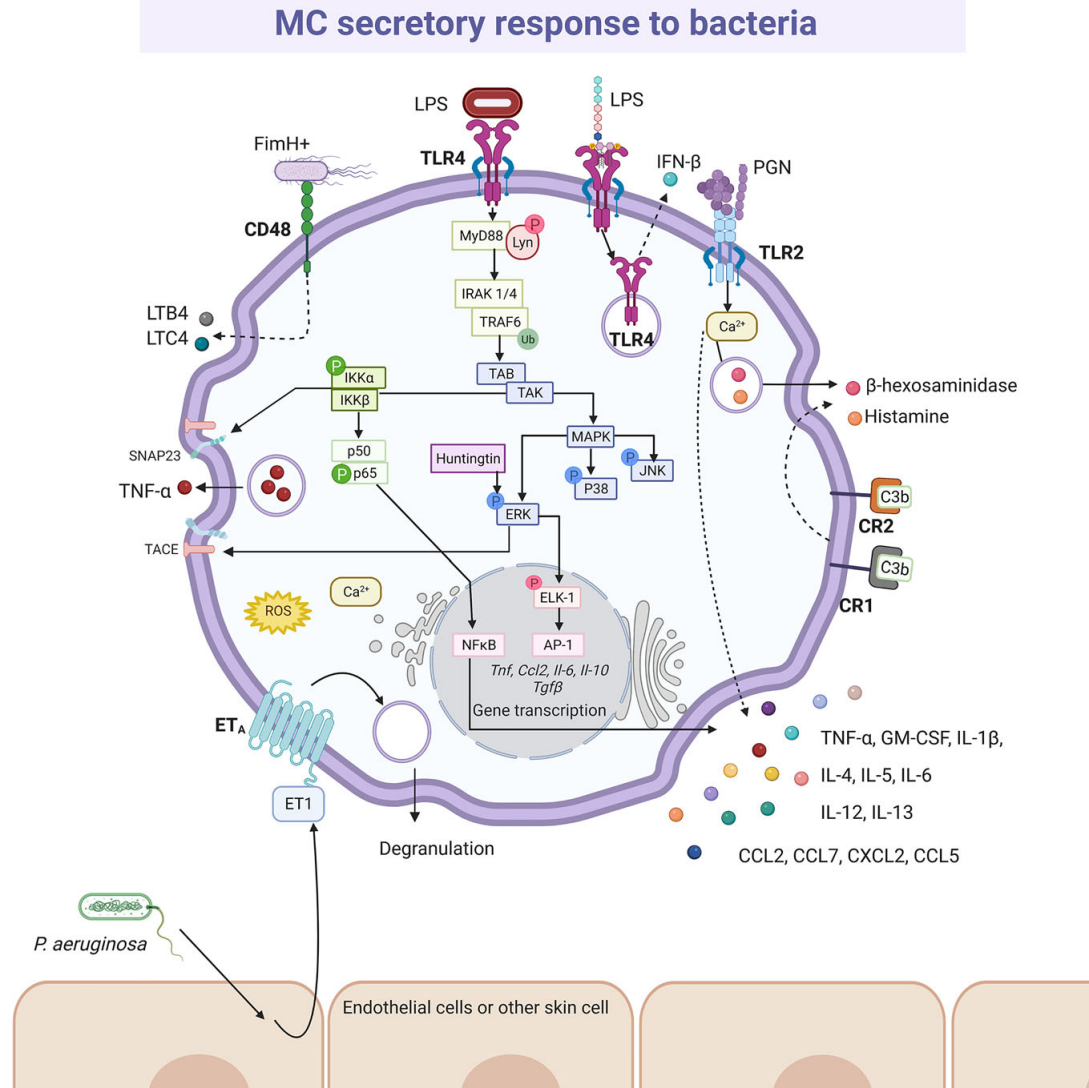
MC-released mediators and signaling pathways elicited by bacteria. After recognition by specific pattern recognition receptors (i.e. TLR4 or TLR2) or specialized receptors (i.e. CR1, CR2 and CD48), distinct signaling cascades are activated in MCs causing the synthesis and secretion of numerous pro-inflammatory mediators, such as leukotriene B4 (LTB4), leukotriene C4 (LTC4), tumor necrosis factor (TNF)-α, interleukins (IL)-4, IL-5, IL-6, IL-12, IL-13, granulocyte and monocyte colony stimulating factor (GM-CSF), and preformed mediators, such as β-hexosaminidase and histamine. The better-known signaling pathway activated by bacteria is the MyD88-dependent cascade leading to cytokine gene transcription after NFκB and AP-1 activation, that requires classical IKK and MAPK (ERK1/2, P38 and JNK) phosphorylation, together with the recruitment of Huntingtin. IKK also contributes to TNF secretion through the phosphorylation of SNAP23 and the ERK1/2-dependent TACE activation. Still controversial, TLR4 is internalized upon LPS recognition and translocated to acidic endo-lysosomes, inducing IFN-β releasing. A particular mechanism has been described for *Pseudomonas aeruginosa*, that promotes endothelin (ET)-1 release from surrounding cells, triggering ETA receptor in MCs. In this scheme solid-lines indicate known pathways and dashed-lines show reported effects of receptor triggering in cases where signaling cascades have not been described. LPS, lipopolysaccharide; PGN, peptidoglycan.

Bacterial activation of MCs is accomplished by a variety of stimuli (**Figure 4**). Gram-positive bacteria such as *Streptococcus equi* (165), or peptidoglycan from *S. aureus* (166) directly activated the cell through TLR2 receptor, although the participation of heterodimers composed by TLR2 and other members of the TRL family of receptors was not evaluated in the mentioned studies. Moreover, Gram-negative bacteria, such as *E. coli*, seemed to trigger TLR4 by its interaction with lipopolysaccharide (LPS) (166), or through CD48 *via* FimH protein (167). Mycobacteria, such as *Mycobacterium tuberculosis*, caused CD48 aggregation and histamine secretion (168). On the other hand, complement proteins were essential in MCs activation during bacterial infections (169), mainly through the CD21/CD35 (CR2/CR1) receptors (170). In addition, *P. aeruginosa* mediated indirectly skin MC activation by the cutaneous production of endothelin-1, a protein that induces MC degranulation through ET_A_ receptors (171, 172). Nevertheless, it is important to highlight that after cell activation the mediators released are not always the same. Thus, BMMCs co-cultured with alive *S. equi* secreted high levels of chemokines such as CCL2/monocyte chemotactic protein (MCP)-1, CCL7/MCP-3, CXCL2/macrophage inflammatory protein (MIP)-2α, CCL5/RANTES (regulated upon activation normal T-cell expressed and secreted), IL-4, IL-6, IL-12, IL-13 and TNF-α. The release of these mediators was activated by stimulation of TLR2 receptor and was dependent on cell-to-cell contact. Under those conditions, although cytokine release was significant, cells showed a reduced degranulation with a low release of histamine (165). Nevertheless, activation of BMMCs through TLR2 receptor by peptidoglycans from *S. aureus* led to calcium mobilization and cell degranulation as well as *de novo* synthesis of cytokines such as TNF-α, IL-4, IL-5, IL-6, and IL-13, but not IL-1β (166). On the other hand, activation of BMMCs through TLR4 by LPS from *E. coli* did not induce degranulation or significant calcium release, although it triggered the *de novo* synthesis of cytokines such as TNF-α, IL-1β, IL-6 and IL-13 after activation of kappa-light-chain-enhancer of activated B cells transcription factor (also known as nuclear factor κB, NFκB) (166). Since heterodimerization of TLR1 or TLR6 with TLR2 has been demonstrated in other cells with distinct consequences on signaling pathway activation (173, 174), further investigation is needed to gain insight into the detailed activation mechanisms of MCs by bacterial products through TLR receptors.

Evidence have shown that *in vitro* exposure of MCs to FimH-expressing *E. coli* generated a high release of LTB4 and LTC4 (175). Thus, the administration of a potent pharmacological LT-synthesis inhibitor reduced the differences in neutrophil influx and bacterial survival induced by intraperitoneal injection of *E. coli* between MC-deficient and MC-proficient (wild-type and MC-deficient but reconstituted) mice. Moreover, MCPT-6(-/-) mice, that lack the protease homologous to human tryptase β-1, lost their ability to eliminate *K. pneumoniae* from the peritoneal cavity; highlighting the role of this protease in the innate immune response against bacteria. That phenomenon was associated with early extravasation of neutrophils to the peritoneal cavity (176). Supporting these results, mouse MCPT-6 triggered the release of CXCL-2/MIP-2 from endothelial cells, a cytokine equivalent to human IL-8 that enhances the release of TNF-α from MCs (177, 178). Additionally, complement activation was essential in MC activation in response to bacterial infection. Particularly, C3 was associated with MC degranulation, TNF-α production, neutrophil infiltration, and bacterial elimination in the CLP model in C3-deficient mice (169). The anaphylatoxin C3a is a potent activator of connective tissue-type MCs, although C3a and related peptides are also shown to inhibit FcϵRI activation in mucosal-type MCs (179). Besides, C3b and C3bi mediate opsonin-dependent phagocytosis in MCs (111, 115), and C3d can activate MCs through CD21/CD35 (170). As human skin MCs can produce C3, process that can be up-regulated by various cytokines (180), and both tryptase and chymase can cleave C3 (181, 182), the participation of locally produced C3 in MC response to bacterial infection requires deeper investigation. Other MC-mediators have been implicated in antibacterial response. BMMCs co-cultured with macrophages inhibited the uptake and growth within macrophages of the Gram-negative bacteria *Francisella tularensis*. Both MC-deficient mice and IL-4R(-/-) mice showed greater susceptibility to infection with *F. tularensis* compared to normal animals, which point out their beneficial roles; although results showed that IL-4 is not mainly produced by MCs in pulmonary infection by *F. tularensis* (183). On the other hand, MC-derived IL-6 improved mice survival following *K. pneumoniae* lung infection and sepsis (184). In line with these results, it was demonstrated the important role of MCs in the healing of skin wounds infected with *P. aeruginosa*; specifically, MCs protected mice from skin infection by secreting IL-6 that induced anti-bacterial effects on keratinocytes by up-regulating the production of AMPs (185). Moreover, it was demonstrated *in vitro* that *M. tuberculosis* activated cultured MCs, triggering the release of preformed mediators such as histamine and β-hexosaminidase, and newly synthesized cytokines such as IL-6 and TNF-α (168). Concerning proteases, the mouse MCPT-4 was associated with the protective role of MCs during urinary tract infections caused by uropathogenic *E. coli* and during the female lower genital tract infections caused by group B *Streptococcus* (GBS) in mice models (186, 187); in the first infectious condition by directly cleaving and activating caspase-1 that induced the death and shedding of bladder epithelial cells and in the last one by cleaving the host extracellular matrix protein fibronectin that diminished GBS adherence.

More recently, the antibacterial activity of β-hexosaminidase was described. MC-deficient mice reconstituted or not with MCs without β-hexosaminidase (β-hexosaminidase(-/-) MCs) presented greater severity in symptoms and a higher rate of death due to intraperitoneal infection with *Staphylococcus epidermidis*, as compared to wild-type mice and MC-deficient mice reconstituted with β-hexosaminidase(+/+) MCs (188). Nevertheless, β-hexosaminidase absence did not change serum allergen-specific IgE levels neither lung infiltration of inflammatory cells in asthmatic animals (188). On the other hand, *in vitro* bacterial growth was inhibited with the addition of β-hexosaminidase(+/+) MCs lysate, but not with that of β-hexosaminidase(-/-) MCs. The authors suggested that β-hexosaminidase together with lysozyme act by destroying the cell wall of *S. epidermidis via* degradation of peptidoglycans (188). However, the microbicidal effect of MC-derived β-hexosaminidase cannot be extrapolated to other Gram-positive bacteria, as no effect was observed on *S. aureus* (188).

The existence of canonical PRR-triggered signal transduction cascades leading to NFκB and activator protein-1 (AP-1) transcription factors and the production of ROS (observed in macrophages and DC) has been confirmed in MCs and explains *de novo* synthesis of cytokines after challenge with bacterial products; in addition, distinctive pathways coupling PRRs to the secretion of pre-formed mediators seem to be quite specific for MCs (**Figure 4**). For example, triggering of TLR4 receptor led to the engagement of the myeloid differentiation primary response 88 (MyD88)-dependent signaling cascade that includes the activation of downstream molecules such as the TNF receptor associated factor 6 (TRAF6) and the IκB kinase (IKK) together with the nuclear translocation of p65 NFκB (166, 189). However, the TLR4-induced TIR-domain-containing adapter-inducing interferon-β (TRIF)-dependent signaling pathway leading to the secretion of IFN-β, whereas broadly observed in macrophages and DC, was reported absent in MCs (190). The absence of this pathway is controversial, since recently, BMMCs showed to release IFN-β after TLR4 induction *via* LPS and the internalization and translocation of the receptor to acidic endo-lysosomal compartments was a prerequisite for cytokine release (191). On the other hand, particular roles of IKK and the mitogen-activated kinase (MAPK) extracellular receptor kinase (ERK)1/2 were found in BMMCs activated through the TLR4 receptor, since those kinases participated in the piecemeal secretion of TNF-α through the phosphorylation of SNAP23 (soluble N-ethylmaleimide sensitive factor attachment protein receptor-23) and the activation of the disintegrin/metalloprotease ADAM-17/TNFα-converting enzyme (TACE), respectively (192, 193). Also, Ca^2+^ mobilization and activation of Lyn and Fyn kinases occurred in BMMCs after LPS-dependent TLR4 triggering (154, 189, 192). Finally, recent evidence indicated that the multifunctional protein Huntingtin was required for the activation of the ERK1/2-AP-1 axis after TLR4 triggering in BMMCs, contributing to the accumulation of TNF-α, IL-6, IL-10 and transforming growth factor (TGF)-β mRNAs and secretion of those cytokines (194).

Regarding NOD-like receptors, although no particular signaling molecules were described in MCs and seems that the formation of inflammasomes and activation of NFκB follows the same pathways that those reported in other immune cells (105, 108), it was shown that those receptors were inducible in response to cathelicidin LL-37 and defensin hBD-2 (108) and were important for MC-microbe interactions leading to exocytosis of mediators. For example, the NOD2-specific agonist muramyl dipeptide promoted TNF-α secretion from MCs and, *in vivo*, a significant increase in NOD2 positive MCs was reported in colonic mucosal biopsies from Crohn´s disease patients compared to those coming from ulcerative colitis or control biopsies (195).

#### Virus

**Figure 5:**
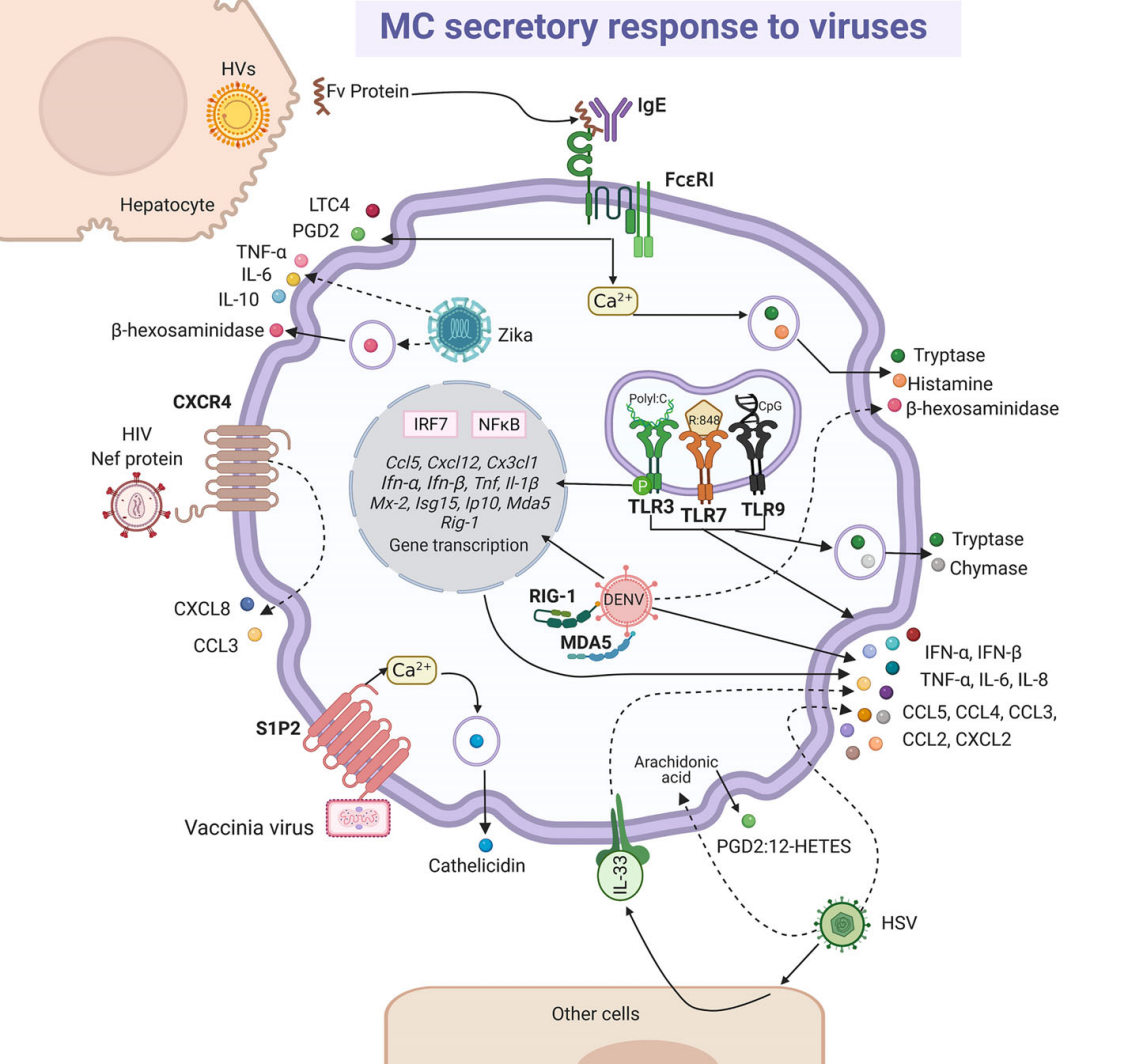
MC-released mediators and signaling pathways in response to viruses. Some viral particles are recognized directly by membrane receptors, i.e. vaccinia virus binds sphingosine-1-phosphate 2 (S1P2) receptor and human immunodeficiency virus (HIV) to CXCR4, triggering signaling pathways leading to cathelicidin or CXCL8 and CCL3 chemokines release, respectively. Intracellular dengue virus (DENV) is probably recognized by RIG-1 and MDA5 and herpes simplex virus (HSV) directly or through the release of alarmin IL-33 by other cells lead to the secretion of cytokines and chemokines, together with the arachidonic acid derivatives prostaglandin 2 (PGD2) and 12-hydroxyeicosatetranoic acid (12-HETES). Fv endogen superantigen from hepatocytes infected by hepatitis viruses (HVs) promotes MC degranulation and the release of leukotriene C4 (LTC4) and prostaglandin D2 (PGD2) by a mechanism that seems to depend on the activation of FcϵRI receptor and calcium mobilization. Zika virus infection promotes MC degranulation and cytokine secretion. Finally, classical responses to viral compounds *via* TLR3, TLR7 and TLR9 receptors have been observed in MCs, that lead to the synthesis of interferon (IFN)-α and IFN-β through the activation of interferon regulatory factor (IRF)-7 and NFκB, and also to the release of tryptase and chymase. Solid-lines indicate known pathways and dashed-lines show reported effects of receptor triggering or MC-virus interactions, although specific signaling cascades remain to be described.

MCs present a diverse response against viruses (196). Studies on the pathogenesis of viruses in their natural hosts have increased our understanding about what happens in humans. In this regard, we can find many similarities in bovine respiratory syncytial virus (RSV) infection and its human homologous hRSV (197). Although, histopathological findings showed degranulation of MCs during infection by bovine RSV (198, 199), using *in vitro* models it was suggested that degranulation was indirectly induced by hRSV (200). The role of MCs on airway hyperreactivity was studied in the onset of viral infection in guinea pig, since it is a feasible model that resembles the observed signs in humans (201, 202). Parainfluenza virus 3 induced degranulation and histamine release in pulmonary MCs from guinea pig, which may represent a significant mechanism to provoke wheezing and asthma pathogenesis (202). Additionally, viral components can stimulate the synthesis and release of *de novo* mediators alone or in combination with degranulation (**Figure 5**). The extracellular version of protein Nef expressed in the early phase of infection of the human immunodeficiency virus (HIV) triggered the release of CXCL8/IL-8 and CCL3/MIP-1α through the CXCR4 receptor in MCs (203). Besides, the indirect activation of MCs during viral infection was documented. In patients affected by acute and chronic viral hepatitis B, C, A and E, the endogen superantigen Fv is produced in high concentrations by hepatocytes, and it induced the secretion of LTC4 or PGD2, as well histamine or tryptase, presumably by interacting with the variable domain of the IgE heavy-chain (204, 205). Although many of these mediators can contribute under certain circumstances to the physiopathology of viral infections, in this section we will focus on the data that have contributed to position the MCs as crucial elements of defense against viruses.

*In vivo* and *in vitro* murine models defined that vaccinia virus triggers MC degranulation by activating S1P2 receptor after binding of lipids of the viral membrane, generating the release of cathelicidin that abolished the virus infectivity (206). In this context, MC activation was dependent on the fusion of the virus envelope to cell membrane. In young mice susceptible to atopic dermatitis (AD), MC-derived cathelicidin was a determining factor to avoid eczema vaccinatum in response to vaccinia virus (207). In this regard, as vaccination with vaccinia virus is contraindicated in AD patients, to define the role of MC-derived cathelicidin will allow to establish better strategies to prevent adverse reactions (207). The antiviral activity of AMPs was demonstrated against human influenza A virus (208), hRSV (209), Zika virus (210) and HIV (211). Concerning dengue virus (DENV), it was observed that DENV infection up-regulated the transcription of CCL5/RANTES, CXCL12, CX3CL1/fractalkine, TNF-α and IFN-α in RBL-2H3 cells (212). Besides, human MC cell lines infected with the DENV in the presence of specific antibodies selectively released chemokines such as CCL3/MIP-1α, CCL4/MIP-1β, CCL5/RANTES, but not IL-8 or CXCL5 (213). These mediators might be involved in the mobilization of lymphocytes, or other immune cells, which favors the early response against the virus. In a recent study, using a cell line of human mature MCs directly exposed to DENV in an antibody-independent manner, it was evidenced that the virus does not replicate in MCs but triggers its degranulation, the synthesis of tryptase, chymase, PGs and LTs and up-regulates the transcription of genes associated with the antiviral response and the Th1-polarization (214). On the other hand, murine intradermal infection with the herpes simplex virus (HSV)-2 induced the synthesis of IL-33 by keratinocytes, that in turn activated the synthesis of TNF-α and IL-6 by MCs, key cytokines in reducing the severity of the infection (215). The same protective effect was mediated by MCs in HSV-1 infection on the cornea; however, in this immune privileged environment the MCs controlled inflammation and viral replication by reducing the infiltration of polymorphonuclear cells (additional reservoirs of the HSV-1), probably due to changes in levels of chemoattractant (216). Thus, authors described that MC-deficient mice showed a decrease in the PGD2:12-hydroxyeicosatetraenoic acid (12-HETES) ratio, and while PGD2 suppresses neutrophil chemotaxis and endothelial transmigration during acute inflammation, 12-HETES is a potent neutrophil chemoattractant that promotes increased vascular permeability. The increased expression of CXCL2/MIP-2α in the corneas of MC-deficient mice might be also facilitating the neutrophil influx during HSV-1 infection. Recently, it was shown that the human placental MCs and HMC-1 cell line were permissible to *in vivo* and *in vitro* Zika virus infection, respectively; in HMC-1 cells, viral infection triggered degranulation as well as the release of TNF-α, IL-6, IL-10, which might induce an optimal defense against the pathogen; however, the pro-inflammatory environment coupled with the viral replication in placental MCs suggest a role of the cell in vertical transmission (217). Then, many questions remain to be resolve about the role of MCs in defense against Zika virus.

Regarding receptors involved in MCs response to viruses, the cytosolic receptors participate in the increased expression of TNF-α and IL-1β, as well as type I IFNs, such as IFN-β and Mx-2, as shown by BMMCs infected with the vesicular stomatitis virus (VSV) (118). It is important to mention that type I IFNs play critical roles in innate host defense against viral infections (218), since after binding to their receptors they activate the expression of hundreds of genes that promote an “antiviral state” in cells (219). Transcripts for MDA5 and retinoic acid-inducible gene-1 were found up-regulated after the infection of MCs with DENV (212, 220) and with VSV, leading to the synthesis of IL-6, IFN-β and IFN-α during VSV infection (221). The activation of the cell by viruses was also dependent on the TLR pathways (222). Activation of TLR3, TLR7 and TLR9 by their respective ligands, polyI:C (double-stranded (ds)RNA analog, TLR3 agonist), R:848 (synthetic TLR7 agonist), and CpG oligodeoxynucleotide (unmethylated consensus DNA sequences, TLR9 agonist), respectively, did not trigger degranulation, but induced the production of TNF-α, IL-6, CCL5/RANTES, CCL3/MIP-1α and CXCL2/MIP-2 by murine fetal skin-derived MCs but not by murine BMMCs (223). Besides, a recent study showed that the stimulation of cultured human peripheral blood-derived MCs (PBMCs) with polyI:C or R848 induced MC activation and the release of chymase, tryptase, IL-8, CCL3/MIP-1α and CCL4/MIP-1β (224), highlighting the diverse functionality of MCs depending on their location and origin. In this context, cultured human PBMCs produced IFN-α through TLR3 in response to RSV, reovirus type 1 and polyI:C, but not TNF, IL-1β, IL-5 or granulocyte-macrophage colony stimulating factor (GM-CSF) (225). The phosphorylation of TLR3 was demonstrated in murine MCs in response to Newcastle disease virus, causing antiviral response mediated by interferon stimulated gene 15 (ISG15), IFN-β, CXCL10/IP-10 and CCL5/RANTES, which was a MC-degranulation independent process (226).

#### Parasites

**Figure 6:**
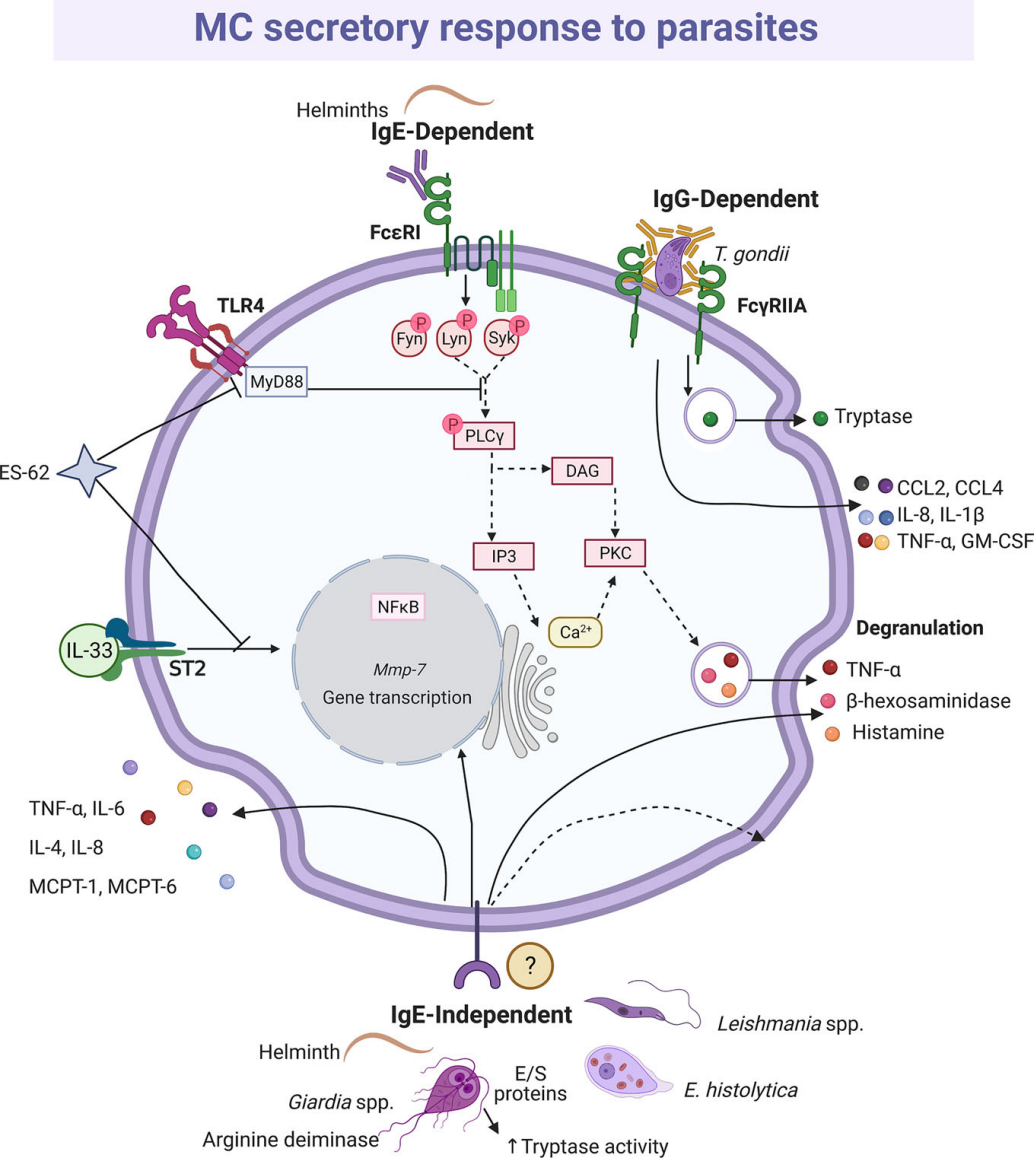
MC-released mediators and signaling pathways elicited by parasites. Distinct parasites promote IgE-dependent and IgE-independent activation patterns. When recognized by IgE, helminths induce full degranulation and cytokine secretion as it has been described for IgE/antigen complexes and the shown intracellular signaling cascade is inferred. Antigen-dependent crosslinking of the IgE molecules bound to FcϵRI monomers causes the activation and autophosphorylation of two Src family kinases, named Lyn and Fyn. In turn, those kinases phosphorylate the immunotyrosine-activation-motifs located in the γ and β subunits of the receptor, creating docking sites for the amplifying kinase Syk. Once recruited and activated, Syk phosphorylates membrane adapters that will conform two main protein complexes directing the signaling i) to the main events leading to calcium mobilization and degranulation, and ii) to secondary processes that contribute to sustain degranulation and induce migration and cytokine production. In order to trigger degranulation, the activated phospholipase C (PLC)γ hydrolyses phosphatidylinositol 4,5-bisphosphate to produce diacylglycerol (DAG) and inositol 3-phosphate (IP3). Those messengers activate several isoforms of protein kinase C (PKC) and the IP3 receptor located in endoplasmic reticulum intracellular Ca^2+^ storages. The main final consequences of this signaling branch are the release of Ca^2+^ to the cytoplasm and the phosphorylation of distinct proteins involved in the fusion of granules to the plasma membrane. Crosslinking of FcγRIIA receptors by bound-cell IgGs results in a polarized and sustained release of the granule content at the contact surface between both cells, named antibody-dependent degranulatory synapse (ADDS). ES-62 protein inhibits interleukin (IL)-33-dependent ST2 receptor activation and targets MyD88, which causes downregulation of cytokine synthesis triggered by TLR4 and FcϵRI receptors, while excretion/secretion (E/S) proteins from *Giardia* increase tryptase activity. IgE-independent activation is mediated by not well-defined receptors and causes histamine and cytokine secretion. In this figure, solid-lines indicate reported effects of receptor triggering or MC-parasite interactions, whereas dashed-lines show suggested activated pathways, assuming the activation of the high affinity IgE receptor (FcϵRI) in this cell type.

Mucosal and connective tissue MCs play important roles in defense against intestinal parasitosis, as it has been reported in infections with *Trichinella spiralis* (227, 228), *Strongyloides ratti* (229, 230) and *Toxocara canis* (231), among others (232, 233). In addition, the MCs seem to play a crucial role in the decrease in the fertility rate of *Heligmosomoides polygyrus* (234). One of the most important MC activation mechanisms in the immune response to parasites is mediated *via* FcϵRI and Fcγ receptors and anti-parasite-specific IgE and IgG antibodies. This fact was demonstrated to *H. polygyrus*, *Nippostrogylus brasiliensis*, *Strongyloides venezuelensis* and *T. spiralis* infection using IgE(-/-), IL-4(-/-) null mice or MC-deficient mice infected with the parasite in the presence or absence of parasite immune sera-derived IgE or IgG (235, 236). In addition, different models of MC-deficient mice showed that MCs play a more important role during the early phase of primary immune response than in the late phase or the secondary response against helminths (230, 237–239). Protection against *Fasciola hepatica* was associated with infiltration of eosinophils, IgE positive cells and MCs (240). The importance of parasite-specific IgE in the protective response to helminths was evidenced by the impaired protective activity in animals with high levels of non-specific IgE that compete for Fc receptors in MCs (241). In this context, degranulation, and histamine production, together with the release of distinct lipid mediators and cytokines was shown in studies where the interaction of MCs and parasites was addressed and the participation of IgE antibodies was identified (reviewed in 242). The pattern of secreted mediators and changes in MC morphology (i.e. degranulation) indicates that the full signaling cascade of FcϵRI, which has been characterized in response to allergens, is activated by parasites (235) (**Figure 6**). On the other hand, tachyzoites of *Toxoplasma gondii* opsonized with IgG specific to the SAG-1 surface antigen and co-cultured with MCs induced a polarized degranulation mediated by ADDS that resulted in tryptase-dependent parasite death. In addition, MCs were activated to produce CCL2/MCP-1, CCL4/MIP-1β, CXCL8/IL-8, GM-CSF, IL-1β and TNF-α (146).

IgE-independent MC activation mechanisms are not underestimated in parasitic diseases. Direct contact with alive *Leishmania* promastigotes induced degranulation of BMMCs, with the release of β-hexosaminidase and TNF-α as well as *de novo* synthesis of the latter (243). *Giardia intestinalis* trophozoites and their total soluble extract increased tryptase expression and IL-6 and TNF-α production by a hybrid rat MC line, and the histamine secretion by peritoneal MCs (244); while the total soluble extract activates the release of IL-6 and tryptase, but not degranulation by BMMCs (245). In addition, it was identified that arginine deiminase from *G. intestinalis*, maybe directly or through its metabolic product citrulline, triggered the release of IL-6 and TNF-α (246). Arginine deiminase is an immunodominant antigen that has been identified *in vivo* and *in vitro* after infection by the parasite (247–249). *Giardia intestinalis* infection induced mRNA expression of MC-derived proteases in intestinal tissue of mice. Besides, MMP-7 was one of the most up-regulated genes and together with NO played a key role in the decline of *Giardia* trophozoites. As MMP-7 is responsible for the production of α-defensins in mice, the protective effect of MCs might be mediated by this AMP (250). Whether the cellular source of MMP-7 was MC or another cell it needs to be elucidated. Interestingly, mature adult mice with deletion in chymase MCPT-4 gene (MCPT-4-/-) showed a significant weight reduction due to *G. intestinalis* infection, a characteristic clinical sign of the symptomatic giardiasis, as compared to MCPT-4+/+ mice; the weight loss was not observed in MCPT-4-/- or MCPT-4+/+ young mice (251). However, one of the proteases that becomes more important in defense against helminths is MCPT-1, since in its absence the intestinal permeability was blocked, affecting the expulsion mechanisms of *T. spiralis* (252). Additionally, experiments in MC-deficient mice suggested that the expulsion of the parasite was dependent on MC-derived IL-4 and TNF-α (253). Moreover, MC proteases were responsible for degrading the collagen-like proteins in the *Necator americanus* cuticle (254). However, as aforementioned, the diversity of parasites and the complex nature of their antigens generate a broad range of responses in the cells. For example, the secretory products of *Entamoeba histolytica* promoted the synthesis of IL-8 by MCs *via* a protease activated receptor-2 independent mechanism (255).

Interestingly, the interaction between parasites and MCs can also lead to the blockage of mediator secretion in this cell. For example, the ES-62 protein, secreted by the parasitic worm *Acanthochilonema viteae*, exhibited immunomodulatory activities lowering MC responsiveness (256). It was found that ES-62 inhibited the signaling from the IL-33/ST2 receptor independently on the phenotype of MCs. Interestingly, ES-62 sequestered MyD88 and then contributed to the downregulation of cytokine expression triggered by TLR4 and FcϵRI receptors (257). On the other hand, parasites may also modulate the activity of MCPTs. In this context, excretory-secretory proteins from *Giardia* increased the enzymatic activity of human and mouse tryptase (245).

#### Fungi

Although it is estimated that 1 billion people worldwide have some type of fungal infection (258), just a little is known about the release of mediators by MCs upon their activation by fungi. Concerning fungal PRRs, the C-type lectin receptor family member Dectin-1 and Mincle (macrophage inducible Ca^2+^-dependent lectin receptor) are expressed in MCs and their signaling systems seem to induce the secretion of pro-inflammatory mediators (259, 260). Curdlan, a Dectin-1 agonist, led to histamine release and degranulation, but not to the production of CCL2/MCP-1, IL-6 or LTC4 (261). On the other hand, Mincle seems to interact with γ and β subunits of the FcϵRI receptor, activating Syk tyrosine kinase and leading to anaphylactic degranulation as observed with IgE/Ag complexes (262).

**Figure 7:**
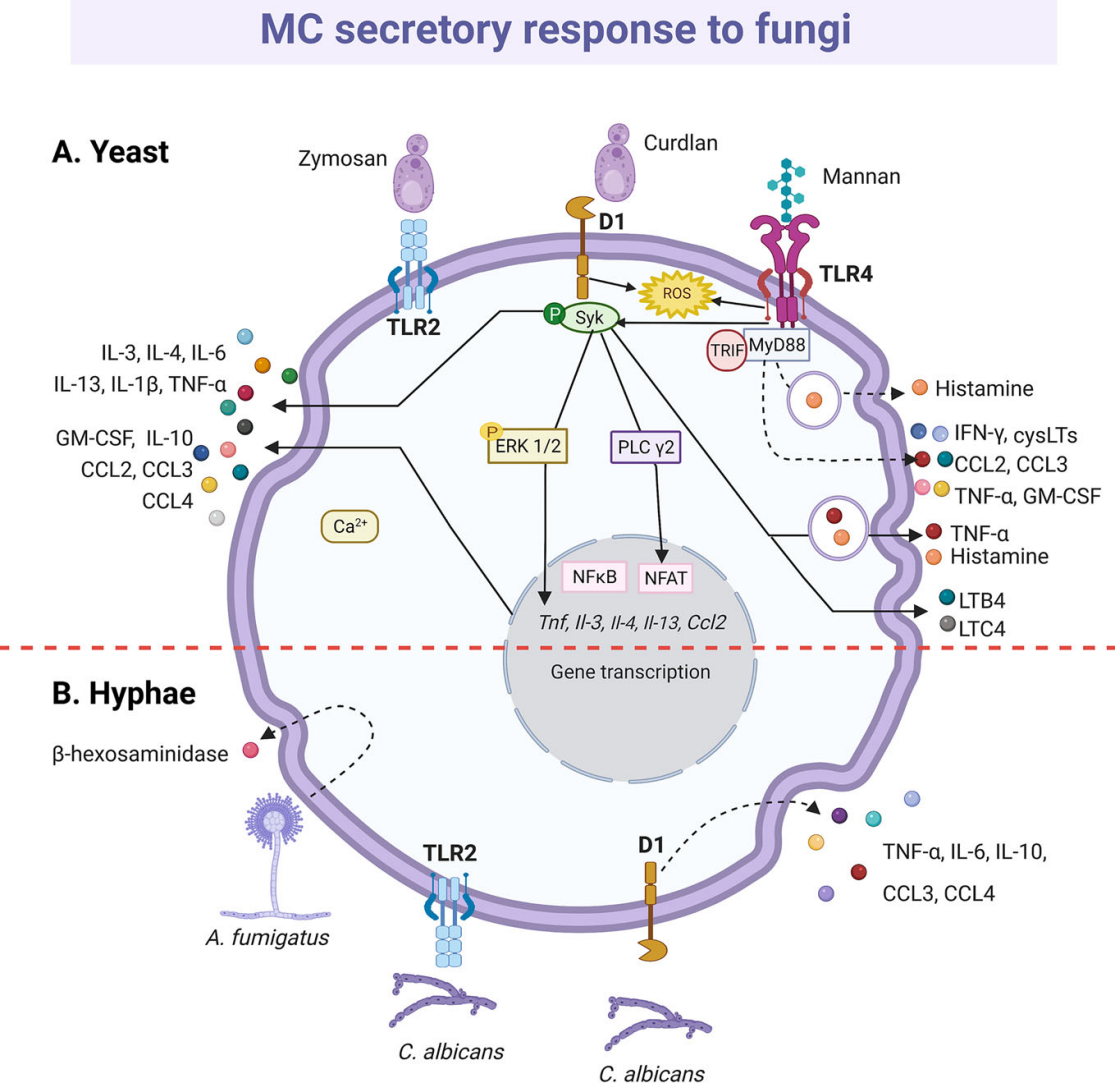
MC-released mediators and signaling pathways in response to fungi. **(A)** Toll-like receptor (TLR)-2 and Dectin-1 (D1) receptors recognize yeasts. Triggering of D1 receptor leads to Syk kinase activation and the release of histamine and cytokines, such as tumor necrosis factor (TNF)-α, interleukin (IL)-1β, IL-3, IL-4, IL-6, IL-10, IL-13 chemokines such as CCL2, CCL3 and CCL4 and granulocyte and monocyte colony stimulating factor (GM-CSF). Secretion of leukotriene (LT)B4 and LTC4 also has been described, together with the reactive oxygen species (ROS)-dependent activation of NFκB. Mannan recognition through TLR4 receptor induces histamine release and ROS, cysLTs, cytokine and chemokine production in a MyD88-, TRIF- and Syk-dependent manner. **(B)** Hyphae also seem to be recognized by TLR2 and D1 receptors that leads to cytokine secretion. Finally, *Aspergilius fumigatus* induces the release of β-hexosaminidase. Dashed-lines show unknown pathways activated in MC response to yeast and hyphae. Solid-lines show fragments of signaling pathways that have been experimentally demonstrated and dashed-lines show reported effects of receptor triggering or MC-fungi interactions. Central red dashed-line separate what is known about the interactions with the yeasts or hyphae of fungi.

Dectin-1 (261, 263) and TLR2 (264) are the receptors mainly involved in the MC antifungal response, which becomes relevant considering that MC is the cell type with the higher expression of Dectin-1 in the skin (259). Zymosan possess β-glucans that are recognized by Dectin-1; however, zymosan can also interact with other receptors due to its complex composition, including heterodimers of TLR1 or TRL6 with TLR2 (265). Therefore, to analyze the specific activation of Dectin-1, ligands such as curdlan are used (**Figure 7**). In RBL-2H3 cells, curdlan triggered MC degranulation (261) and caused the phosphorylation of phospholipase Cγ2 and the expression of IL-3, CCL2/MCP-1, IL-13, IL-4 and TNF-α mRNAs in a Syk dependent manner, as the effect was abrogated when cells were preincubated with the Syk inhibitor R406 (263). Remarkably, curdlan-induced cytokine mRNAs, such as TNF-α and IL-3 were also sensitive to the MAPK/ERK kinase inhibitor PD98059, showing that several downstream proteins, such as ERK1/2, are shared between Dectin-1 and FcϵRI in MCs (263). Besides, zymosan induced *de novo* synthesis of LTs, GM-CSF and IL-1β by CBMCs in a dose-dependent manner (264). In human MCs, LTC4 was released in a Syk-dependent mechanism *via* Dectin-1 receptor (266); meanwhile, zymosan induced the generation of intracellular ROS through Dectin-1, and to a lesser extent *via* TLR2, in murine BMMCs (267). BMMCs also released IFN-β in response to zymosan *via* TLR2; where the internalization of the receptor and the endosome maturation were needed (191). Recently, the antifungal response of MCs through TLR4 receptor was demonstrated. Rat peritoneal MCs stimulated with mannan released histamine and produced cysLTs, ROS and pro-inflammatory cytokines and chemokines, such as IFN-γ, GM-CSF, TNF-α, CCL2/MCP-1 and CCL3, *via* TLR4 and dependent on MyD88, TRIF and Syk (268). Mannan also increased the gene expression of different immunoregulatory and pro-inflammatory cytokines and the chemoattraction of MCs. Interestingly, cell response to mannan was enhanced in IgE-sensitized MCs (268), which is important to be considered in the context of IgE-mediated allergic conditions, as ongoing fungal infection in humans could exacerbate and worsen the course of the allergic disease.

The release of mediators by MCs in response to dimorphic fungal pathogens can be different depending on their morphotype (yeast or mycelia) and state of maturation. Degranulation of RBL-2H3 cells was induced by *Paracoccidioides brasiliensis* yeasts and by mature *Aspergillus fumigatus* hyphae, but not by their immature hyphae or conidia (269, 270). Furthermore, a recombinant version of the PbPga1 protein from the yeast surface of *P. brasiliensis*, activated the release of IL-6 *via* NFκB (269). *C. albican*s also induced degranulation and *de novo* synthesis of various cytokines by MCs, although results are still controversial. Nieto-Patlán et al. reported that both yeasts and hyphae induce the production of TNF-α, IL-6, IL-10, CCL3/MIP-1α and CCL4/MIP-1β by BMMCs *via* Dectin-1, without ruling out TLR2 involvement; while IL-1β was only induced by yeast cells (271). Nevertheless, De Zuani et al., using the same MC type, showed that only yeasts triggered the release of TNF-α, IL-6, IL-13, and IL-4 (272). Likely, during the early response, *C. albicans* extracellular destruction is mediated by products derived from the cell degranulation, such as histamine and TNF-α (113, 144, 271, 273), since the MC fungicidal activity was greater to extracellular than to engulfed yeasts (113, 144).

The MC response to *Sporothrix schenckii*, a dimorphic fungus that causes a chronic subcutaneous mycosis called sporotrichosis that affects both humans and animals, was also studied. Both *S. schenckii* conidia and yeast induced TNF-α and IL-6 secretion by peritoneal MCs without a significant degranulation, and while the former potentiated histamine secretion induced by C48/80, the latter activated MC through ERK1/2 pathway (274, 275). BMMCs also dose-dependent released IL-6, TNF-α, IL-1β and IL-10 in response to *S. schenckii* yeasts (276). Although IL-6 and TNF-α are cytokines that play important roles in the defense against fungi (277–279), it is suggested their participation in the pathogenesis of *S. schenckii* infection, and this injurious side of the MCs will be discussed later.

#### Modulation of Innate and Adaptive Response to Infection

Through the release of mediators, the MCs establish connections with various cells at the site of infection, supporting the microbicidal activity of macrophages and neutrophils. In addition, MCs participate in the chemoattraction of various pro-inflammatory cells to site of infection. During infection by Gram-negative bacteria, the production of TNF-α, LTs and mouse MCPT-6 participated in neutrophil chemoattraction (159, 160, 175, 176). Additionally, *de novo* production of TNF-α and GM-CSF by MCs was implicated both in the recruitment of neutrophils and the improvement of their phagocytic activity and ROS generation in a model of acute lung inflammation induced by LPS (280). Furthermore, MC-derived GM-CSF decreased neutrophil spontaneous apoptosis (280), and MC-derived IL-6 improved bacterial killing by neutrophils (184). Studies performed in histidine decarboxylase(-/-) mice and infected with *M. tuberculosis* showed that MC-derived histamine mediated the production of TNF-α and IL-6, as well as suppressed the Th1 response, prompting an inflammatory pathology (281). On the other hand, during viral infection, MCs usually produce a series of chemokines that modulate the migration of cells associated to antiviral activity. The chemoattraction of NK and NKT cells in a MC-dependent fashion at the site of DENV infection was associated with MC expression of CCL5/RANTES, CXCL12, CX3CL1/fractalkine, TNF-α and IFN-α (212). While the production of CXCL8/IL-8 by CBMCs after exposure to mammalian reovirus serotype 3 led to the chemoattraction of NK cells (282). In helminth infection, mouse MCPT-6 was associated with eosinophil chemoattraction in an IgE-dependent manner (283). In addition, HMC-1 cells infected with *C. albicans* induced the recruitment of neutrophils, probably due to the increase in IL-8 synthesis (144). Interestingly, a recent study in MC-deficient mice showed that MCs participate in the resolution of zymosan-induced inflammation by promoting the efferocytosis mediated by macrophages, possibly through IL-4 and CXCL1 secretion (191).

In the context of the adaptive immune response, the products secreted by MCs recruit DC precursors, promote the influx of monocyte-derived DCs, activate DCs for antigen presentation and induce their mobilization to draining lymph nodes. In response to peptidoglycans or Gram-positive bacteria, MCs activated skin Langerhans cells, which leads to an increase in the number of these cells at the draining lymph nodes (284). It is known that histamine favors the capture of antigens, the cross-presentation of DCs, the expression of costimulatory molecules by DCs and the induction of Th-differentiation profiles (285, 286). Thus, the histamine secreted during infection by activated MCs might be modulating DC response. In this sense, the histamine receptor (HR)2 expressed by DCs was involved in the attraction of plasmacytoid DCs to draining lymph nodes in response to the pathogen (284). Furthermore, the administration of MC-derived exosomes containing exogenous antigens and heat shock proteins to naive mice enhanced specific humoral responses and induced phenotypic and functional maturation of DC both *in vivo* and *in vitro* (287). Likewise, MC granules exocytosed in response to LPS were captured intact by dermal DCs, promoting the maturation and migration of DC to the lymph nodes and improving the priming of T cells; the TNF embedded in exocytosed MC granule was partially responsible for these effects (288). Besides, TNF released by MCs in mice infected with *E. coli* increased the expression of E-selectin in local blood vessels, facilitating the recruitment of DC to the site of infection (289). Furthermore, activation of murine MCs through TLR3-polyI:C induced CCL5/RANTES, CCL4/MIP-1β and keratinocyte-derived chemokine production, triggering the recruitment of CD8^+^ T lymphocytes (226). MCs also interact directly with CD8^+^ T lymphocytes by presenting antigen *via* MHC molecules class I, and induce CD8^+^ T lymphocytes to produce IL-2, IFN-γ, and CCL3/MIP-1α. At the same time, they regulate the cytotoxic activity of CD8^+^ T lymphocytes by increasing their degranulation and up-regulating granzyme expression. This effect is enhanced when MCs are activated *via* PRR, by LPS or polyI:C (290). The presentation of antigens to CD4^+^ T lymphocytes by MCs is not ruled out, since *in vitro* stimulation of murine MCs with LPS and IFN-γ or *in vivo* with LPS or *Leishmania major* induced the expression of MHC class II and costimulatory molecules (103). The just-mentioned *in vitro* experiments showed that MCs *via* MHC II can reactivate antigen-experienced CD4^+^ T lymphocytes and antigen-specific T regulatory (Treg) cells, over naïve T cells. In this sense, it was demonstrated using longitudinal intravital multiphoton microscopy and DC/MC double reporter mice, that after cell-to-cell contact DCs transferred class II MHC proteins to dermal MCs in the context of skin inflammation (291); although this DC-to-MC communication exacerbated the subsequent T-cell driven skin inflammation and promoted T cell survival, more studies are needed to clarify the physiological impact of this phenomenon. Finally, there is a cross-communication between MCs and Treg lymphocytes. The adoptive transfer of *in vitro*-stimulated CD4^+^ CD25^+^ Treg cells to mice with polymicrobial sepsis increased the number of peritoneal MCs and the production of TNF-α, in addition to improving bacterial elimination and animal survival (292). In addition, histamine released by BMMCs activated by FcϵRI cross-linking inhibited the suppressive activity of CD4^+^ CD25^+^ Treg cells through the HR1 receptor, probably due to the reduction in the expression level of CD25 and Foxp3 (293). Despite the discoveries made so far concerning MC-Treg intercommunication (294–296) there are still many questions to be resolved in the setting of the antimicrobial response.

## Detrimental Roles of Mast Cells During Antimicrobial Response

Different studies support that under a high microbial load in the body, the uncontrolled secretory response of MCs can contribute to the development of a pathological conditions. In this sense, while MCs showed a protective role in CLP mice models that caused moderate peritonitis, the MC response was detrimental in severe peritonitis with a high bacterial load, leading to an increase in animal mortality (297). Using MC-deficient mice (*W^sh^/W^sh^*) intraperitoneally engrafted with either wild-type MCs or TNF-deficient MCs, it was shown that MC-derived TNF contributes to the deleterious effects of MCs after severe CLP induction or after intraperitoneal inoculation of *S. typhimurium*. In these experimental conditions, MCs might be susceptible to activation by bacteria carried within the blood stream, and the resulting release of mediators could potentially have lethal effects on the host as they quickly reach the blood vessels due to perivascular location of MCs (298), resulting in severe systemic effects. Accordingly, when animals with CLP were administered with the MC stabilizer sodium cromoglycate clinical manifestations of sepsis were attenuated and there was an improved mice survival by preventing splenocyte apoptosis and the consequent increase in serum levels of the high mobility group box-1 alarmin, suggesting that MCs contribute to systemic inflammation during sepsis (299). The functional importance of MC systemic degranulation during infection was evaluated by compartment-specific MC reconstitution in *W^sh^/W^sh^* mice with CLP-induced septic peritonitis. This study demonstrated that while MC reconstitution only at the peritoneal cavity improved the survival of animals, MC reconstitution both at the peritoneal and systemic levels decreased animal survival (300). In addition, systemically reconstituted animals with IL-6(-/-) BMMCs improved survival compared to those reconstituted with IL-6(+/+) BMMCs, suggesting that degranulation and IL-6 release from MCs located distant to the site of infection play a detrimental role during CLP-induced infection (300). A later study described a potential mechanism of indirect harmful participation of MCs during severe peritonitis, which was mediated by the early release of preformed IL-4, achieving immunosuppressive effects on the ability of macrophages to phagocytose bacteria (301). A similar double-face behavior of MCs has been described in DENV infection. Localized MC response to DENV might protect the host by recruiting key cells involved in virus clearance and by limiting the number of cellular targets to viral infection (212, 302). On the other hand, granule particles released extracellularly by virus-infected skin MCs contained DENV and could disseminate and propagate the infection in mice through lymph (303). This newly proposed mechanism of virus spreading is in accordance with the described interaction between DENV envelope proteins and heparin (304). Concerning dengue pathology, the MC participation in the vascular loss induced during viral infection in severe states of disease was reported. In experimental models of systemic DENV infection using a virus CI, MC mediators able to modulate vascular endothelium, such as the mice chymase MCPT-1, were elevated in serum (305). Chymase levels were also increased in serum of dengue fever and dengue hemorrhagic fever patients as compared to healthy controls (305). Two indicators of vascular leaking, dye leakage into tissues and hematocrit levels, were decreased in MC-deficient mice, and recovered after MC reconstitution. Besides, this study confirmed the involvement of MCs and LTs in dengue-induced vascular permeability using the MC-stabilizing compound cromolyn and ketotifen and the antagonist of LT receptor montelukast (214, 305). Besides chymase and LTs, MC-derived serotonin was also recently implicated in thrombocytopenia in a severe model of dengue-induced disease (306); thus, the potential of MCs as a therapeutic target to limit dengue vasculopathy or thrombocytopenia should be evaluated in clinical trials. According to results in peritonitis and DENV infection models, while local and immediate MC activation during infection seems to be beneficial, sustained, and systemic activation may not be.

In tuberculosis, it is speculated that TNF-α released by MCs might play a role in the formation of the mycobacterial granuloma, which results in latent disease that can be reactivated later in life (115, 307). A correlation between MCs number and granuloma formation has been described. Analyzing lymph nodes from patients with tuberculous lymphadenitis, MC number was positively correlated with the number of granulomas and the number of multinucleated giant cells (308). The data about MCs in leprosy, a chronic dermato-neurological granulomatous disease caused by *Mycobacterium leprae*, are controversial. Most of the studies indicate an increased number of MCs in skin biopsies of lepromatous lesions, in comparison with other leprosy forms (309–311), except for one study in which a higher dermal MC number was found around granulomas in skin biopsies from patients with tuberculoid or mild-borderline leprosy in comparison to lepromatous leprosy biopsies (312). A more recent study showed that there is a greater amount of degranulated versus intact MCs and a predominance of tryptase positive versus chymase positive MCs in the skin of leprosy patients, independently of leprosy form and reactional episodes (313). These data suggest that MC derived mediators can perpetuate inflammation during *M. leprae* infection, and MC tryptase might be exerting detrimental effects on tissue structure and remodeling in leprosy lesions, as it has mitogenic activity on fibroblasts and increases type I collagen production (69). In support of this notion, an association between collagen increase and tryptase-rich MC density in the epineurium of leprous nerves was described (314). Whether MC response contribute to immunity or disease pathogenesis in chronic granulomatous diseases remains to be deeply studied.

Data also suggest that MCs develop harmful roles during antimicrobial response when the infection is associated with a pre-existing inflammatory disorder. Skin colonization with *S. aureus* was associated with worsening of the inflammatory process linked to AD (315). Among *S. aureus* exotoxins, δ-toxin can activate MC degranulation in an IgE- and allergen-independent manner (316). In experimental models of AD, mice colonized with wild-type *S. aureus* developed higher IgE levels and a more severe inflammatory skin disease than mice inoculated with the bacterium deficient in δ-toxin. Strikingly, in MC-deficient mice (*W^sh^/W^sh^*) inoculated with the wild-type *S. aureus* the level of IgE and the intensity of skin inflammation induced by epicutaneous sensitization was decreased in comparison with wild-type mice, but the severity of the skin disease was restored upon adoptive transfer of MCs into the skin of *W^sh^/W^sh^* mice (316). As different studies show an indispensable role of MCs in the pathogenesis of experimental AD induced by epicutaneous sensitization (317, 318), these results suggest that MC activation by *S. aureus* in the setting of AD exacerbates the pre-existing inflammatory and atopic process. However, more research is needed in this field as it was also suggested protective effects or no participation of MCs in spontaneous AD-like disease or inflammation developed by genetically modified mice (319, 320). *M. sympodialis* infection is also related to the exacerbation of the inflammatory response in AD. MCs responded to *M. sympodialis*, but the response was higher when cells were obtained from patients with AD than those derived from healthy donors (259). *Malassezia* extract induced the production of LTs by sensitized and non-sensitized MCs, the degranulation and production of CCL2/MCP-1 by sensitized cells, as well as improved IgE-dependent degranulation and impaired the synthesis of IL-6 *via* TLR2/MyD88. These changes in the MC response induced by *M. sympodialis* might cause an exacerbated inflammatory response in patients with AD (260). Similarly, MCs are implicated in the pathogenesis of gastritis. An increased MC density was found in mucosa biopsy from subjects with gastritis, and the number was even higher in *Helicobacter pylori*-infected gastric mucosa specimens (321). While MCs in *H. pylori*-infected gastric mucosa showed degranulation, no findings of degranulation were seen in the normal stomach (322). These data suggest that MC response to *H. pylori* infection might be exacerbating the inflammatory response underlying gastritis, as a positive correlation between MC density and intensity of inflammation was described (321). According to all these studies, MC hyperactivation by recurrent infections in the context of an inflammatory disorder can exacerbate pathological tissue damage.

MCs also play crucial roles in the pathogeny associated with some infectious diseases, such as that caused by viruses. It was described that the gp120 glycoprotein of HIV-1, characterized as a superantigen that interacts with the heavy chain of IgE, triggers the release of proinflammatory, angiogenic and lymphangiogenic mediators from human lung MCs (323). As serum IgE levels were elevated in subjects with HIV infection compared to controls (324, 325), this study was the first approach to decipher the possible involvement of MC mediators in chronic lung diseases, that are prevalent among HIV patients (326–328). Besides, human MC progenitors can be HIV infected and retain the virus with their maturation (329). MC participation as a virus reservoir is of great impact on pathology as they are long-lived cells, abundant at viral replication sites and chemoattracted in response to HIV antigens, resistant to the virus cytotoxic effects, and able to contribute to HIV transmission (330–332). In this line, MC precursors cultured *in vitro* from fetal or adult CD34^+^ progenitors co-expressed CD4, CXCR4, and CCR5 and were susceptible to R5 tropism in viral infection, but only marginally susceptible to X4-HIV infection. When IgE-FcϵRI aggregation was induced by HIV gp120 or antigen from *Schistosoma mansoni* eggs, the expression of CXCR4 in MC precursors was up-regulated, increasing their susceptibility to X4 and R5X4 virus infection (333). These data suggest that HIV-positive individuals with pre-existing comorbid conditions associated with elevated levels of IgE, such as atopic diseases or helminth infections, may predispose to a predominant X4 virus phenotype, which has been associated with a more rapid progression to AIDS in infected individuals (334). In the same context of viral infections, it was reported that the activation of brain MCs was causative of worsening infection, morbidity, and mortality in a mice model of Japanese encephalitis virus infection (335). MCs are resident immune cells in the central nervous system that are strategically located near the blood-brain barrier and the neurovascular unit (336). Particularly, MC chymase was identified as the key mediator involved in the increase of permeability in the blood-brain barrier that promotes Japanese encephalitis virus neuroinvasion and neurological dysfunction (335). In addition, MC-deficient mice (*W^sh^/W^sh^*) exhibited resistance to inflammatory disease induced by influenza A virus infection, suggesting that the histamine, LTs, cytokine and chemokine secreted by cultured MCs upon influenza A virus infection might be contributing to the excessive host immune response against the virus (337). Similarly, MC-deficient mice (both *W^sh^/W^sh^* and *Sl/Sl^d^*; the latter harbors deletions in the SCF coding region) showed reduced myocardial inflammation and necrosis, accompanied by an increase in animal survival, compared to normal mice after infection with the encephalomyocarditis virus. Histopathological severity of the myocardial lesions induced by the virus was significantly increased in MC-reconstituted animals, which indicates that MCs are participating in the pathogenesis of viral myocarditis (338). Besides viral diseases, MCs have been also implicated in the development of other infectious pathologies. As previously mentioned, MCs activated by yeast of *S. schenckii* secrete cytokines, mainly TNF-α and IL-6 (275, 276). Nevertheless, when tissue fungal dissemination was evaluated in rats infected with the fungus, the absence of functional MCs in the inoculation site reduced fungal dissemination and the setting of a more severe sporotrichosis (274). The MC contribution to sporotrichosis was recently corroborated using models of MC-depleted mice, and *Sporothrix* virulence was linked to MC cytokine production and the latter to disease activity in patients with sporotrichosis (276).

MCs have been described as potential reservoirs for different pathogens. *S. aureus* promoted its internalization within skin MCs during infection to avoid the extracellular antimicrobial activities (132). *S. aureus* responded to stress imposed by extracellular antimicrobial weapons released by MCs by up-regulating α-hemolysin and other fibronectin-binding proteins. The former was involved in *S. aureus* internalization within MCs (339). Particularly, the interaction between bacterial α-hemolysin and ADAM10 of MCs and the subsequently activated signaling induced the up-regulation of β1-integrin expression on MCs, which mediated *S. aureus* internalization through a pathway different from the normal phagocytic one. Bacterial α-hemolysin was also involved in bacterial survival within the MCs (339). Through hiding within MCs, staphylococci not only avoid clearance but also establish an infection reservoir that could contribute to a chronic carriage. In the same context, it was shown that *E. coli* was up-taken by mice BMMCs in antibody deficient conditions upon FimH-CD48 interaction through a mechanism mediated by caveolae (120). In macrophages, internalized *E. coli* by FimH employing a similar caveolar endocytic pathway showed an increased intracellular survival as compared to opsonized bacteria internalized *via* antibody (340), which suggests that *E. coli* contained in MC caveolar chambers might be also avoiding intrinsic bactericidal activity bypassing phago-lysosomal fusion. However, bacteria viability inside MC caveolae needs a further demonstration, as an interaction of internalized caveolae with lysosomal compartment was described (341, 342).

**Figure 8:**
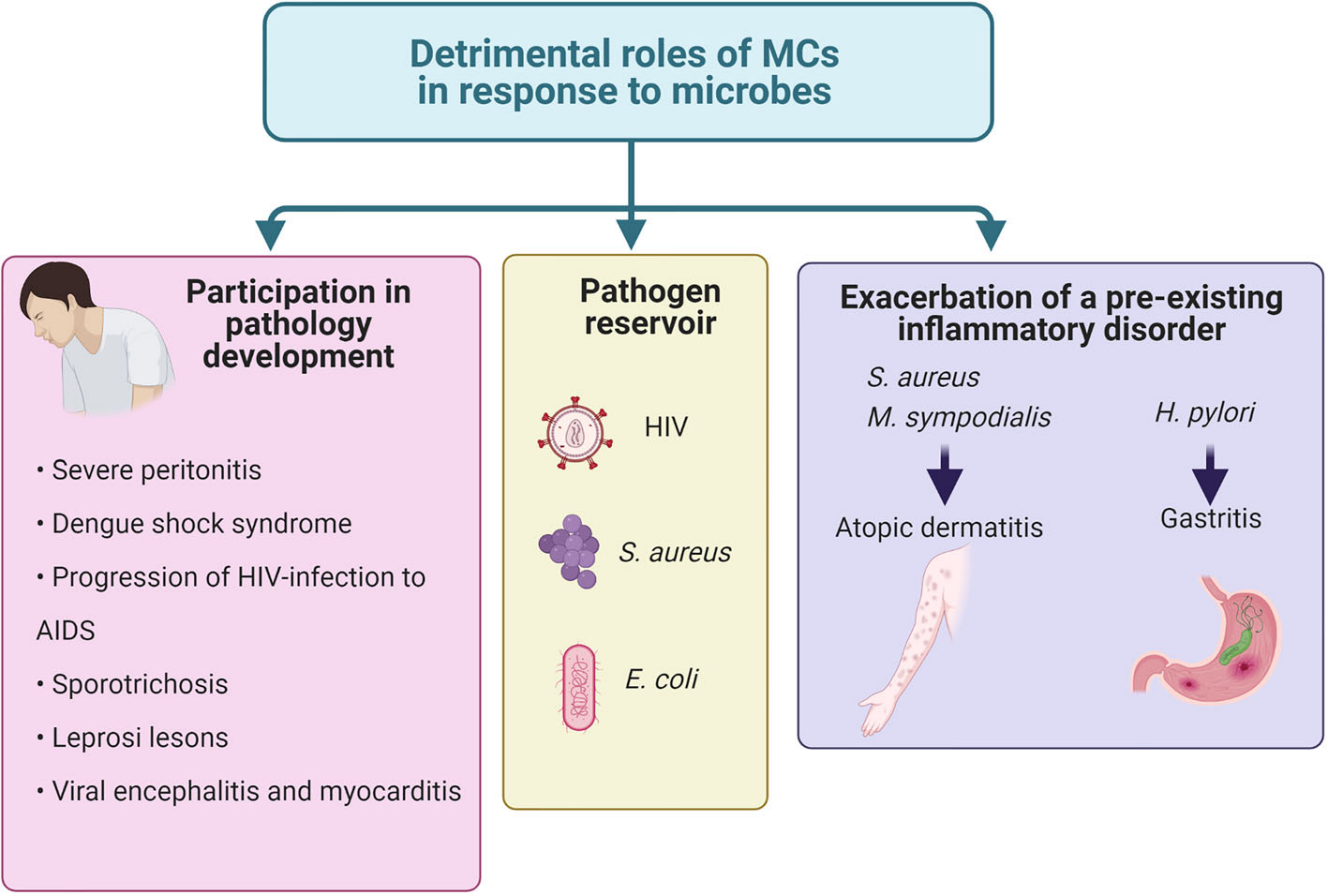
Harmful actions of MCs during infection. MCs have been found to contribute to the worsening of complex pathologies and distinct pre-existing inflammatory conditions. Also, they have been proposed to be reservoirs for distinct virus and bacteria.

The detrimental roles described to MC as a consequence of interaction with microbes are summarized in **Figure 8**; nevertheless, and before closing this section it is worthy to mention that it was reported the first evidence that MC response to an opportunistic pathogen might be associated with allergy onset. Gastrointestinal *Candida* colonization promotes sensitization against food antigens in mice, at least partly due to MC-mediated hyper-permeability in the gastrointestinal mucosa (343). Previous reports had positively associated *H. pylori* infection and the development of food allergy and AD by linking the infectious process with the inhibition of oral tolerance (344–346). Recent works showed that the interaction of *C. albicans* with different MC types, i.e. mucosal or stromal MCs, induced different cytokine microenvironments which contributed respectively to barrier function loss, fungal dissemination, and inflammation or to increase mucosal immune tolerance in gastrointestinal or vulvovaginal candidiasis. The IL-9/MC axis was associated with this dual role of the cell (347, 348).

Finally, few works have suggested the MC participation in the development of both COVID-19 pathology and post-COVID syndrome (349, 350), although more studies are needed to demonstrate the direct implication of the cell in both conditions. An increased MC density was a distinguishing pathological feature in the lungs of COVID-19 patients compared to H1N1-induced pneumonia and control subjects (351), and the levels of chymase, tryptase and carboxypeptidase A3 were higher in serum from SARS-CoV-2 infected patients with generalized inflammation than in uninfected donors (224). Besides, a retrospective cohort study showed that famotidine intake by COVID-19 patients during hospitalization statistically reduced the risk of intubation or death (352). It was suggested that the principal famotidine mechanism of action for COVID-19 was targeting HR2 activity, and that the development of clinical COVID-19 involved dysfunctional MC activation and histamine release (353).

## Conclusions and Perspectives

MCs can respond to parasites, bacteria, viruses, and fungi. They perform different antimicrobial mechanisms, such as phagocytosis, ET formation and the release of granular content or *de novo* synthesized mediators. MC mediators efficiently initiate the recruitment of additional innate effector cells crucial to pathogen clearance, such as neutrophils, monocytes/macrophages, NK cells, NKT cells, or eosinophils. MCs are also associated with the regulation of the adaptive response developed in response to the invading pathogen by directly promoting T-cell activation or by modulating the migration and functionality of DCs.

**Figure 9:**
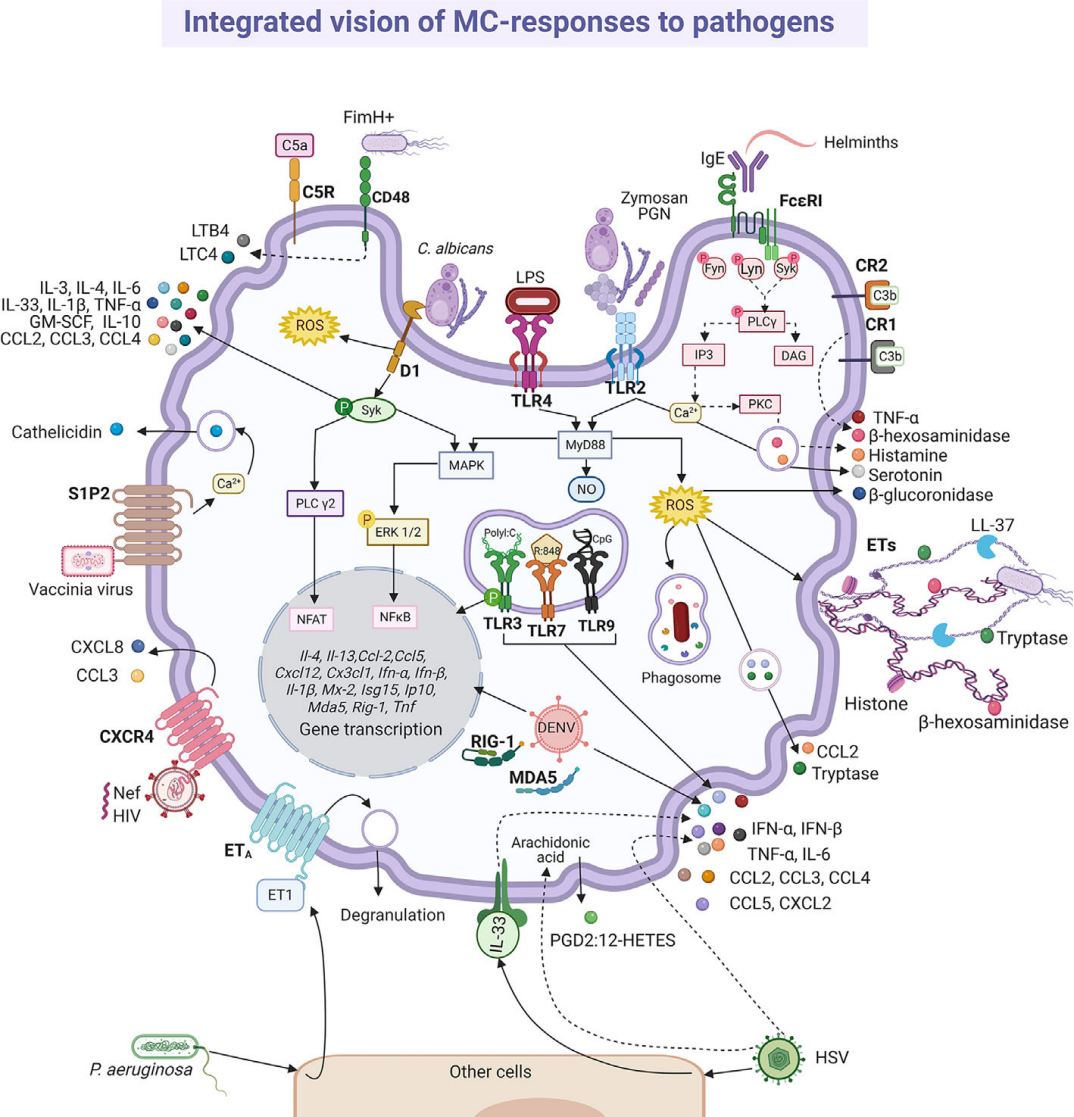
Integrated responses of MCs to distinct pathogens. Distinct pattern recognition receptors (PRRs) expressed in MCs directly recognize pathogens, promoting phagocytosis, extracellular traps (ETs) formation and the release of pre-formed and *de novo* synthesized mediators. Canonical signaling pathways described for PRRs have increased complexity in MCs, where PRR triggering leads not only to the activation of NFκB but also to the secretion of granule content by anaphylactic and piecemeal degranulation. MC activation is observed also as a secondary event after the production of mediators by other cells, which causes the amplification of the initial inflammatory response. On the other hand, IgE-mediated FcϵRI signaling cascade by parasites leads to a protective anaphylactic degranulation response that appears to require the activation of well-described signaling pathways participating in the allergic response. Finally, bacterial phagocytosis, ET release and secretion of mediators seem to be connected by mechanisms to be defined yet. Solid-lines indicate known signaling pathways, whereas dashed-lines indicate suggested pathways or reported effects triggered by receptor stimulation or interactions of MCs with pathogens. LPS, lipopolysaccharide; PGN, peptidoglycan.

However, the wide variety of MC mediators allow multifaceted effects, promoting host defense against pathogens on one hand but inducing damage to the host on the other. The outcome of MC response to pathogens seems to depend on the context in which the cell is activated, being able to entail protection or damage. The main factors involved in this dual role are the followings: *i*) the pathogen distribution, load, and location; *ii*) the compartment or organ in which the activated MC is located; *iii*) the previous existence of a pathological condition associated with the infection; *iv*) the potential use of MCs as a reservoir; and *v*) whether it is an acute or chronic infectious process. More research is needed to complete the signaling pathways described in MCs when responding to pathogen encounters and to identify the points of connection or the distinctive molecules among the pathways involved in phagocytosis, ET release and secretion of mediators (summarized in **Figure 9**). Also, future research should consider the possible pathogen-induced epigenetic changes that chronic infections could induce in MCs, inducing long-term alterations in their phenotype that could modify the response from protective one to detrimental. With that information, it will be possible to suggest potential therapeutic intervention strategies directed not only to generate immune protection or resolve inflammation but also to limit or avoid tissue damage in those infectious scenarios in which the MC plays harmful roles.

The high incidence of infections with fatal outcomes in humans and the goal that we are facing of developing new treatments, as many bacteria have generated resistance to antibiotics (354–360), highlight the importance of generating knowledge about MC response to the infection process. Animal models are mostly used to evaluate the immune response to pathogenic agents as they induce immunological responses homologous to humans, although several differences are present. Therefore, mechanisms demonstrated to MCs during antimicrobial scenario in animals need to be proved to occur in humans, to later propose potential therapies aid to modulate MC activity.

## Author Contributions

MJ and ES conceived the review. MJ, DC-G, LC-D, MP-R, CG-E, and ES wrote de manuscript and designed the figures. MP-R drew the figures. All authors contributed to the article and approved the submitted version.

## Funding

This work was supported by Autonomous University of Aguascalientes (grant PIBB20-1 to ES) and by Conacyt (grant CF-2019-51488 to CG-E).

## Conflict of Interest

The authors declare that the research was conducted in the absence of any commercial or financial relationships that could be construed as a potential conflict of interest.

## Abbreviations

12-HETES, 12-hydroxyeicosatetraenoic acid; AD, atopic dermatitis; ADAM, disintegrin/metalloprotease; AMP, antimicrobial peptide; AP-1, activator protein-1; BM, bone marrow; BMMC, bone marrow progenitor derived mast cell; CBMC, umbilical cord blood derived mast cell; CI, clinical isolate; CLP, cecal ligation and puncture; DC, dendritic cells; DENV, dengue virus; EMP, erythro-myeloid progenitor; ERK, extracellular receptor kinase; ET, extracellular trap; FcϵRI, high-affinity receptor to IgE; GAS, group A *Streptococcus*; GM-CSF, granulocyte-macrophage colony stimulating factor; HIV, human immunodeficiency virus; HR, histamine receptor; HSC, hematopoietic stem cell; HSV, herpes simplex virus; IFN, interferon; IKK, IκB kinase; IL, interleukin; IP3, inositol 3-phosphate; LAT, linker of activation of T cells; LPS, lipopolysaccharide; LTs, leukotrienes; MAPK, mitogen-activated kinase; MC, mast cell; MCETs, mast cell-derived extracellular traps; MCP, monocyte chemotactic protein; MCPT, mast cell protease; MHC, histocompatibility complex; Mincle, macrophage inducible Ca^2+^-dependent lectin receptor; MIP, macrophage inflammatory protein; MMP, matrix metalloproteinase; MyD88, myeloid differentiation primary response 88; NADPH, nicotinamide adenine dinucleotide phosphate; NETs, neutrophils extracellular traps; NFκB, nuclear factor kappa-light-chain-enhancer of activated B cells; NO, nitric oxide; NOD, Nucleotide-binding oligomerization domain; PBMCs, peripheral blood-derived mast cells; PLC, phospholipase C; PGs, prostaglandins; PRRs, pattern-recognition-receptors; RANTES, regulated upon activation normal T-cell expressed and secreted; ROS, reactive oxygen species; RSV, respiratory syncytial virus; SCF, stem cell factor; SNAP23, soluble N-ethylmaleimide sensitive factor attachment protein receptor-23; TACE, disintegrin/metalloprotease ADAM-17/TNFα-converting enzyme; TGF, transforming growth factor; TLR, Toll-like receptor; TNF, tumor necrosis factor; TRAF6, tumor necrosis factor receptor associated factor 6; Treg, T regulatory; TRIF, TIR-domain-containing adapter-inducing interferon-β; VSV, vesicular stomatitis virus.

## Glossary

**Table:**

12-HETES	12-hydroxyeicosatetraenoic acid
AD	atopic dermatitis
ADAM	disintegrin/metalloprotease
AMP	antimicrobial peptide
AP-1	activator protein-1
BM	bone marrow
BMMC	bone marrow progenitor derived mast cell
CBMC	umbilical cord blood derived mast cell
CI	clinical isolate
CLP	cecal ligation and puncture
DC	dendritic cells
DENV	dengue virus
EMP	erythro-myeloid progenitor
ERK	extracellular receptor kinase
ET	extracellular trap
FcϵRI	high-affinity receptor to IgE
GAS	group A *Streptococcus*
GM-CSF	granulocyte-macrophage colony stimulating factor
HIV	human immunodeficiency virus
HR	histamine receptor
HSC	hematopoietic stem cell
HSV	herpes simplex virus
IFN	interferon
IKK	IκB kinase
IL	interleukin
IP3	inositol 3-phosphate
LAT	linker of activation of T cells
LPS	lipopolysaccharide
LTs	leukotrienes
MAPK	mitogen-activated kinase
MC	mast cell
MCETs	mast cell-derived extracellular traps
MCP	monocyte chemotactic protein
MCPT	mast cell protease
MHC	major histocompatibility complex
Mincle	macrophage inducible Ca^2+^-dependent lectin receptor
MIP	macrophage inflammatory protein
MMP	matrix metalloproteinase
MyD88	myeloid differentiation primary response 88
NADPH	nicotinamide adenine dinucleotide phosphate
NETs	neutrophils extracellular traps
NFκB	nuclear factor kappa-light-chain-enhancer of activated B cells
NO	nitric oxide
NOD	Nucleotide-binding oligomerization domain
PBMCs	peripheral blood-derived mast cells
PLC	phospholipase C
PGs	prostaglandins
PRRs	pattern-recognition-receptors
RANTES	regulated upon activation normal T-cell expressed and secreted
ROS	reactive oxygen species
RSV	respiratory syncytial virus
SCF	stem cell factor
SNAP23	soluble N-ethylmaleimide sensitive factor attachment protein receptor-23
TACE	disintegrin/metalloprotease ADAM-17/TNFα-converting enzyme
TGF	transforming growth factor
TLR	Toll-like receptor
TNF	tumor necrosis factor
TRAF6	tumor necrosis factor receptor associated factor 6
Treg	T regulatory
TRIF	TIR-domain-containing adapter-inducing interferon-β
VSV	vesicular stomatitis virus
